# Bound Exciton Complexes in Near-Infrared Emitting Quantum Shells

**DOI:** 10.1021/acsnano.5c18640

**Published:** 2026-01-15

**Authors:** Dulanjan Harankahage, Divesh Nazar, Korneel Molkens, Mykhailo V. Bondarchuk, Christopher M. Hicks, Andrew A. Marder, Michael Montemurri, Adam Roach, Ivo Tanghe, Liangfeng Sun, Richard D. Schaller, Benjamin T. Diroll, Anton V. Malko, Alexander N. Tarnovsky, Dries van Thourhout, Zeger Hens, Pieter Geiregat, Mikhail Zamkov

**Affiliations:** † The Center for Photochemical Sciences, Bowling Green, Ohio 43403, United States; ‡ Department of Physics AndAstronomy, 1888 Bowling Green State University, Bowling Green, Ohio 43403, United States; § Department of Chemistry, 1888 Bowling Green State University, Bowling Green, Ohio 43403, United States; ∥ Department of Physics, 12335 The University of Texas at Dallas, Richardson, Texas 75080, United States; ⊥ Physics and Chemistry of Nanostructures, 26656 Ghent University, Gent 9000, Belgium; # NOLIMITS Center for Non-Linear Microscopy and Spectroscopy, Ghent University, Gent 9000, Belgium; ¶ Center for Nanomaterials, 1291 Argonne National Laboratory, Lemont, Illinois 60439, United States; ∇ Photonics Research Group, Department of Information Technology, Ghent University, Gent 9000, Belgium; ○ Department of Chemistry, 3270 Northwestern University, Evanston, Illinois 60208, United States

**Keywords:** *Auger recombination*, *near-infrared*, *quantum dots*, *photodetectors*, *telecommunications*, *fiberoptics*

## Abstract

Near-infrared (NIR) light sources based on colloidal semiconductor nanocrystals (NCs) represent a scalable, low-cost alternative to epitaxial semiconductor platforms. However, their performance remains hindered by rapid Auger recombination, a problem that is particularly pronounced in narrow-bandgap materials. Here, we report on CdS/HgS/CdS and CdS/HgCdSe/ZnS quantum shells (QSs), a class of spherical quantum wells specifically engineered for a suppression of nonradiative Auger processes. Fabricated QSs exhibit tunable NIR emission with photoluminescence quantum yields reaching ∼60% below 1000 nm and up to 30% near 1300 nm. Optical gain and stimulated emission were observed in CdS/HgS/CdS QSs. In contrast, CdS/HgCdSe/ZnS QSs displayed a photoinduced absorption in lieu of optical gain despite demonstrating a comparatively stronger Auger suppression. Transient absorption spectroscopy revealed that this phenomenon arises from the formation of bound multiexciton complexes that induce long-lived sub-bandgap multiexciton states. The observation of such bound excitonic clusters at room temperature offers a pathway toward nonlinear NIR photonic phenomena, including biexciton–exciton cascade emission, optical modulation, and single-exciton gain.

## Graphical abstract

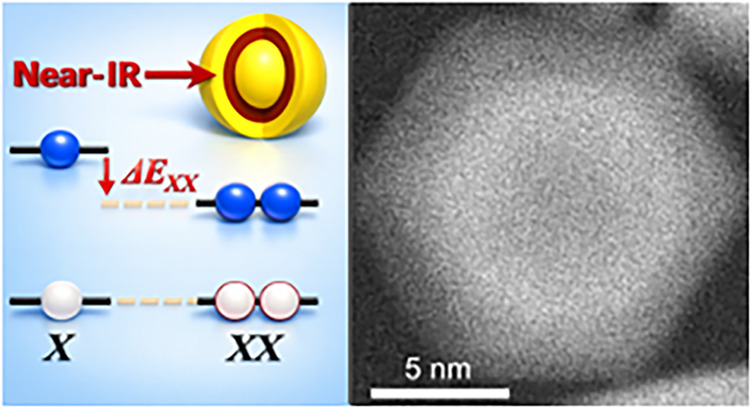

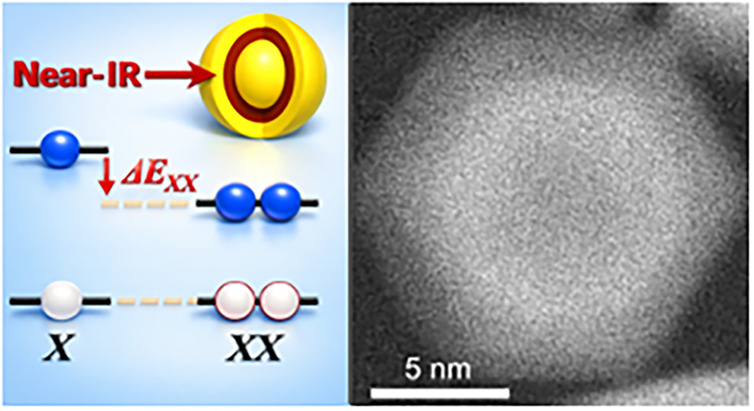

## Introduction

Near-infrared (NIR) light sources are critical for optical telecommunications, bioimaging, and industrial sectors.^1^ Most NIR laser diodes and LEDs are still based on costly epitaxial semiconductors, such as InGaAs and HgCdTe,^2,3^ making them less accessible for mass production. To address this challenge, colloidal semiconductor nanocrystals (NCs) have emerged as a promising low-cost and scalable alternative for NIR applications,^4–7^ offering size-tunable emission, solution-processability, and compatibility with a wide range of substrates.^8^

Light-emitting infrared technologies utilizing colloidal NCs face unique obstacles,^9,10^ one of which is a fast nonradiative Auger recombination. This process is particularly pronounced in narrow bandgap semiconductors,^11^ suppressing the stimulated emission and converting excitation energy into heat, which, in turn, raises the optical gain thresholds. Unlike infrared photodetectors that typically operate at low excitation levels and cryogenic temperatures,^12,13^ infrared lasers are directly affected by the Auger recombination due to high injection current densities and high-temperature operation. This issue is especially notable in commonly used PbS and PbSe NIR colloidal NCs, where nonradiative Auger recombination is enhanced due to an 8-fold band-edge degeneracy.^14^ Recent efforts by the Konstantatos group, however, have made significant strides in overcoming this challenge. By introducing controlled doping to lift the band-edge degeneracy in PbS,^15^ they successfully demonstrated amplified spontaneous emission (ASE) in the 1530–1650 nm spectral region,^15^ achieving lasing thresholds as low as 300 μJ/cm^2^.^16^ These results were further improved through ZnO nanocrystal surface passivation and the development of PbS/PbSSe core–shell architectures.^17^ However, despite these promising strides in threshold reduction, the underlying issue of short gain lifetimes caused by Auger recombination of trion-like multicarrier configurations remains unresolved.^18^

To overcome the high band-edge degeneracy present in lead chalcogenide NCs, recent efforts have shifted toward mercury chalcogenides, such as HgTe and HgSe, and their Cd-based ternary alloys. These semiconductors benefit from a 2-fold band-edge degeneracy and offer broader spectral tunability compared to lead chalcogenides. For instance, 0D-2D colloidal NCs of mercury chalcogenides have shown impressive photoluminescence (PL) quantum yields exceeding 60% at 1700 nm,^19^ and have been incorporated into continuous-wave lasing devices with low thresholds.^5^ Additionally, mercury chalcogenide infrared LEDs have demonstrated electroluminescence extending to λ = 5000 nm,^20,21^ with external quantum efficiencies (EQEs) of 4.5%.^6,22^ Further advantages are associated with their ability to form Cd-based alloys with the direct band gap ranging across the short-wavelength (SWIR) and midwavelength (MWIR) infrared regions, as was demonstrated through the development of NIR LEDs based on Hg_1–*x*_Cd_*x*_Se nanoplatelets (NPLs) and quantum dots (QDs).^23,24^

Several design strategies have been developed to suppress Auger recombination in colloidal nanocrystals. One approach is to engineer graded interfaces that smooth the confinement potential and eliminate sharp band offsets,^25–29^ thereby reducing nonradiative multicarrier decay. A second, conceptually distinct strategy is to increase the nanocrystal volume,^30^ for instance by employing 2D nanoplatelets^31,32^ or bulk-like QDs^33^ (including thick-shell CdS systems^34^), which lowers Auger rates through their characteristic volume scaling. In addition, type-II heterostructures, in which electrons and holes are spatially separated across the interface, can further mitigate Auger recombination by reducing wave function overlap.^35,36^ Recently, a significant suppression of Auger decay in the visible-range emitters was achieved by using semiconductor quantum shells (QSs)^37–48^ (e.g., CdS_bulk_/CdSe/CdS core/shell/shell). These structures, consisting of a spherical quantum well (e.g., CdSe) sandwiched between wider-bandgap core and outer shell components (e.g., CdS), allow for a bright biexciton emission with the corresponding biexciton PL quantum yields approaching 100%.^43^ This has resulted in excellent gain characteristics of QSs in optically pumped lasers,^44–46^ positioning a 2D spherical-shell morphology as a promising architecture for exploring optical amplification in the NIR regime.

Here, we report on the synthesis of near-infrared emitting colloidal quantum shells. These nanostructures incorporate a narrow-gap HgS or Hg_*x*_Cd_1–*x*_Se spherical quantum well, sandwiched between wide-bandgap CdS core and outer shell layers (see Figures 1a and S2a). Both CdS/HgS/CdS and CdS/Hg_*x*_Cd_1–*x*_Se/ZnS QSs exhibit strong photoluminescence below 1000 nm, with PL quantum yield (PL QYs) around 60%. At longer wavelengths (∼1300 nm), however, CdS/Hg_*x*_Cd_1–*x*_Se/ZnS QSs retain a comparatively higher PL QY (∼30%) versus only ∼5% for CdS/HgS/CdS QSs. The advantages of the quantum shell architecture in suppressing Auger recombination were evident through long-lived biexciton states. In case of CdS/Hg_*x*_Cd_1–*x*_Se/ZnS QSs biexciton lifetimes were found to be 160–180 times longer than in compositionally similar Hg_*x*_Cd_1–*x*_Se QDs, indicating efficient spatial separation of carrier wave functions by the shell geometry. The QSs also displayed complex multiexciton behavior under the high-power excitation regime. While optical gain and stimulated emission were observed in CdS/HgS/CdS QSs, brighter-emitting CdS/Hg_*x*_Cd_1–*x*_Se/ZnS QSs displayed photoinduced absorption in lieu of net amplification. Transient absorption spectroscopy attributed this phenomenon to the formation of bound excitonic complexes, analogous to those reported in epitaxial quantum wells at cryogenic temperatures,^49–51^ and in 2D CdSe nanoplatelets at room temperature.^52^ In case of colloidal CdS/Hg_*x*_Cd_1–*x*_Se/ZnS QSs, these multiexciton states induce a pronounced, transient red-shift of the absorption edge, effectively acting as a dynamic bandgap renormalization.^33,53^ Such photoinduced bandgap narrowing paves the way to nonlinear optical applications, including biexciton–exciton cascade emission, optical modulation, and the potential realization of single-exciton gain.

**Figure 1:**
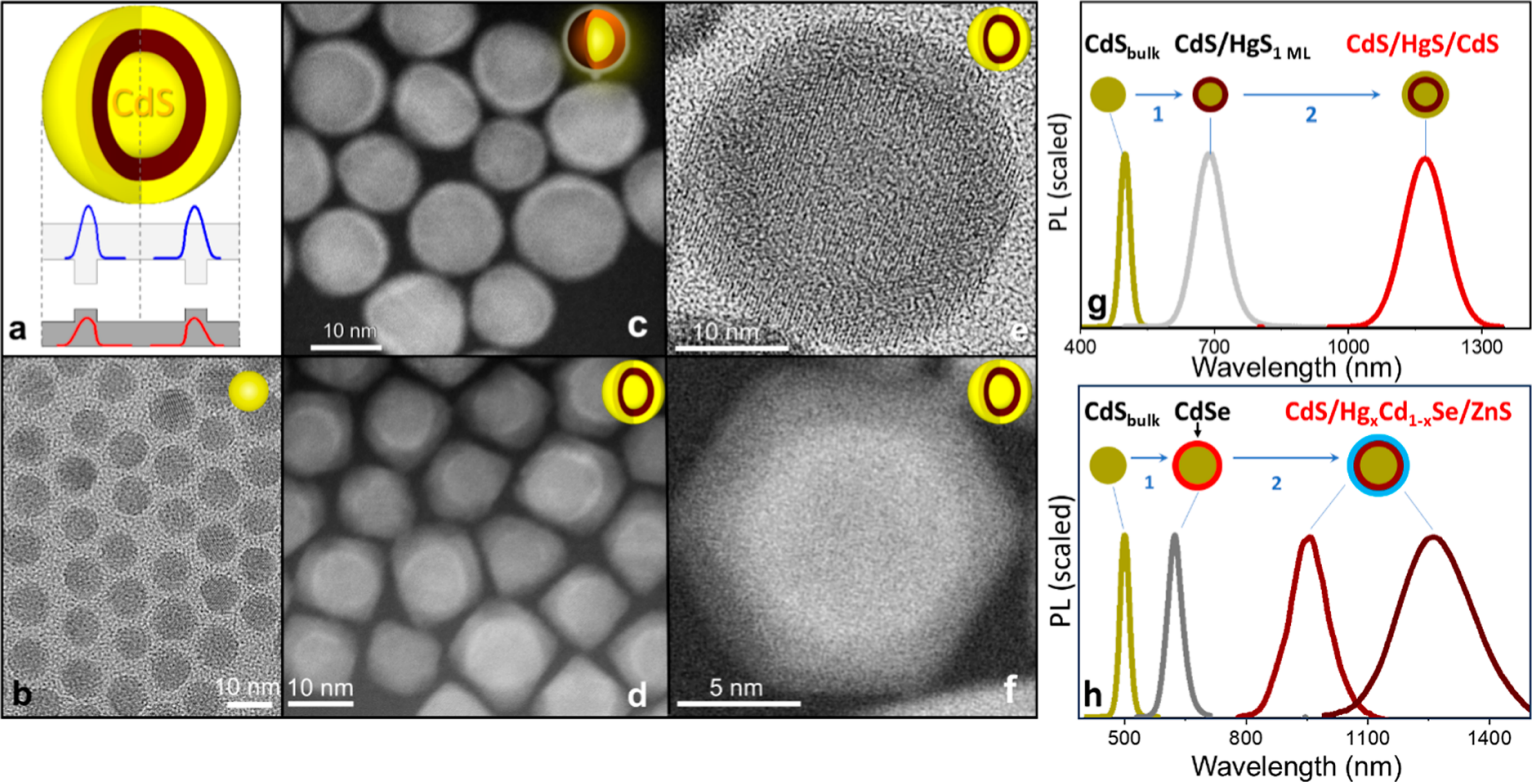
(a) Schematic band diagram of a CdS/HgS/CdS QS, illustrating a narrow-bandgap HgS quantum well confined between wide-bandgap CdS core and shell layers. (b) TEM image of bulk-like CdS core NCs used as a core component of QSs. (c) High-angle annular dark-field scanning transmission electron microscopy (HAADF-STEM) image of CdS/HgS core/shell structures showing the location of surface-grown HgS layer (brighter shade). (d) HAADF-STEM image of CdS/HgS/CdS QSs, highlighting the contrast between the HgS quantum well and the outer CdS layers. (e) TEM image of a representative CdS/HgS/CdS QS, showing the HgS layer as a darker contrast band. (f) High-resolution HAADF-STEM image of a single CdS/HgS/CdS QS. (g) PL spectra corresponding to key synthesis stages of CdS/HgS/CdS QSs: initial CdS cores (7–12 nm), intermediate CdS/HgS core/shell structures formed via controlled Cd-to-Hg cation exchange, and final CdS/HgS/CdS QSs obtained by CdS overcoating. (h) PL spectra corresponding to intermediate phases of the CdS/Hg_*x*_Cd_1–*x*_Se/ZnS QSs synthesis: initial CdS core nanocrystals (7–12 nm); intermediate CdS/CdSe core/shell structures; partially exchanged CdS/Hg_*x*_Cd_1–*x*_Se NCs resulting from Cd-to-Hg cation exchange; and fully formed CdS/Hg_*x*_Cd_1–*x*_Se/ZnS QSs synthesized via low-temperature (130 °C) shell overgrowth.

## Results and Discussion

The geometry of CdS/HgS/CdS QSs can be viewed as a large-core version of CdS/HgS/CdS quantum well quantum dots (QWQDs).^54,55^ By using a “bulk-size” CdS core component, we increase the effective exciton volume in the HgS shell, which leads to the reduction in the Auger recombination rate. Additionally, the presence of a CdS core promotes the formation of the infrared-emitting β-HgS crystalline shell phase,^56^ instead of the α-HgS cinnabar structure (*E*_g_ = 2.0 eV),^57^ which is typically favored in colloidal synthesis. Structurally, the CdS/HgS/CdS configuration benefits from nearly lattice-matched phases of CdS and β-HgS semiconductors, which is expected to minimize strain-induced defects. Furthermore, the stronger covalent bonding of mercury with chalcogens, compared to cadmium, helps in achieving a uniform formation of the HgS layer through a cation exchange process (Cd^2+^ → Hg^2+^).

The synthesis of CdS/HgS/CdS QSs starts with the growth of a 7–12 nm CdS “bulk” core, via the coalescence of smaller CdS nanoparticles in the mixture of oleylamine and CdCl_2_.^58^ During this process, small CdS NCs collide and fuse into larger morphologies featuring thermodynamically defined particle shapes (see Figure 1b). This phenomenon, reported previously,^59–61^ occurs as a result of CdCl_2_ addition,^62,63^ which lowers the melting point of a NC, leading to a thermodynamic reorganization of the lattice. The emission of large-size CdS core NCs was observed at λ ≈ 505 nm (see Figure 1g), which is close to that of bulk wurtzite CdS. Next, Hg^2+^ to Cd^2+^ cation exchange reaction was used to form a thin HgS shell by heating large-diameter CdS core NCs in the presence of HgCl_2_ and oleylamine. To further increase the shell thickness, a secondary HgS layer was grown through simultaneous coinjection of S-ODE and Hg­(OAc)_2_-OLAM at 120 °C, as detailed in the Supporting Information section. The formation of the HgS shell was confirmed by high-angle annular dark-field scanning transmission electron microscopy (HAADF-STEM) in Figure 1c. The increasing thickness of the HgS layer was also accompanied by the characteristic emission changes, with the PL peak gradually redshifting from 600 to 1300 nm (see Figure 1g). We note that if temperatures exceeding 300C° are used at this step, an unwanted transition from β – HgS to α – HgS (*E*_g_ = 2 eV) will occur, causing the PL blue shift to 620 nm (Figure S2).

The fully grown CdS/HgS/CdS QS structures exhibited a near hexagonal nanoparticle shape, as evident from HAADF-STEM images (Figures 1f and S3). Electron microscopy images in Figure 1d confirm the presence of a distinct HgS quantum well layer in CdS_bulk_-HgS-CdS QSs characterized by uniform thickness and some evidence of alloying between the CdS and HgS phases, suggesting a graded interface between the two materials. The X-ray diffraction (XRD) pattern (Figure S4) reveals the characteristic wurtzite structure of the CdS components. However, no clear signatures of the cubic β-HgS phase are observed, which may be attributed to its relatively low volume fraction (∼10%) within the predominantly CdS structure, as well as a possibility of strain at the heterointerface.

The PL QY measurements of CdS/HgS/CdS QSs (excitation at λ = 400–600 nm) in Figure 2b reveal significant differences in performance based on the thickness of the HgS layer. QSs with thin HgS layers, emitting in the 750–900 nm range, achieved PL QY values of 40–60%. Meanwhile, QSs emitting beyond 1100 nm exhibited a significant reduction in PL QY, dropping to 5–12%. These trends were further supported by PL intensity decay measurements. Thin-HgS QSs displayed nearly monoexponential decay, consistent with a minimal trap state contribution (Figure S6a), while thick-HgS QSs showed multiexponential decay containing a very fast, τ ≈ 2 ns initial decay component (Figure 2c). Attempts to mitigate trap state decay through additional shelling were unsuccessful, as they caused alloying at the HgS/CdS interfaces (see Figure S5), resulting in a broader emission and lower PL QY. We note that the fast decay component, observed in thick-HgS QS samples could, in principle, include a contribution from Auger recombination, as commonly observed in multicarrier states. However, the fast decay component seen in Figure 2c was consistently present even in the limit of the low excitation power, < *N*_eh_> ∼0.1, which suggests a more significant contribution from trapping^64^ rather than Auger decay.

**Figure 2:**
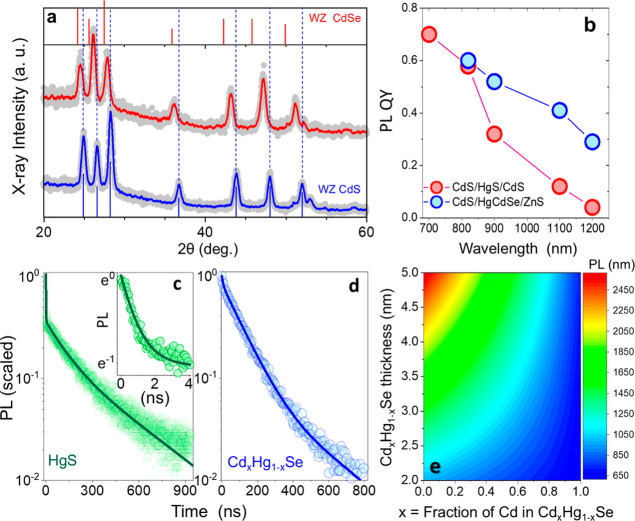
(a). XRD pattern of CdS NCs (blue) and CdS/Hg_*x*_Cd_1–*x*_Se QSs (red) showcasing distinct diffraction peaks corresponding to wurtzite (WZ) crystal structure. (b) PL QY measurements of CdS/HgS/CdS and CdS/Hg_*x*_Cd_1–*x*_Se/ZnS QSs. (c) Time-resolved PL measurements of thick-HgS CdS/HgS/CdS and (d) CdS/Hg_*x*_Cd_1–*x*_Se/ZnS QSs, acquired at low excitation fluences corresponding to < *N*_eh_> < 0.1. (e). Contour plot showing the emission wavelength for the 1S_e_-1S_h_ transition in Cd_*x*_Hg_1–*x*_Se supported 2D nanosheets, plotted as a function of both the composition parameter *x* (the Cd to Hg ratio) and nanosheet thickness, calculated using the data from ref 66.

The close similarity of CdS and β-HgS lattice parameters, while beneficial for minimizing lattice strain, introduces synthetic challenges. We observed that CdS and HgS layers can readily intermix during the reaction, forming a Hg_*x*_Cd_1–*x*_S alloy, shifting the morphology from the desired CdS/HgS/CdS QS to a thermodynamically more stable Hg_*x*_Cd_1–*x*_S form. To overcome this issue, we introduced a moderately strained core/shell structure incorporating a ternary Hg_*x*_Cd_1–*x*_Se spherical shell. The CdS/Hg_*x*_Cd_1–*x*_Se/ZnS QS morphology was inspired by recent advancements on shelling of mercury chalcogenide NPLs.^23,65^ CdS/Hg_*x*_Cd_1–*x*_Se core/shell architecture introduces a moderate, ∼4% lattice mismatch between Hg_*x*_Cd_1–*x*_Se and CdS phases, which effectively suppresses interstitial alloy formation and minimizes Cd/Hg cation interdiffusion. In addition, the Hg_*x*_Cd_1–*x*_Se composition of the spherical shell, in theory, should enable a broader spectral tunability than HgS, with its band gap supporting continuous emission tuning from 600 nm to over 2500 nm (Figure 2e).

CdS/Hg_*x*_Cd_1–*x*_Se/ZnS QSs are grown starting with bulk-sized CdS core NCs (Figure 1b), prepared using an aggregative growth strategy, described above. A CdSe shell was subsequently deposited using a previously established method,^39^ resulting in the onset of visible emission (see Figure 1h). To achieve uniform Cd^2^ → Hg^2+^ cation exchange, the CdS/CdSe NCs were treated with HgCl_2_ dissolved in oleylamine (OLAM). The reaction temperature at this stage was found to be a critical parameter in determining the extent of Hg incorporation into the CdSe shell. In this study, the cation exchange process was conducted at 100 °C to achieve the desired composition for NIR emission.

The deposition of the outer shell on CdS/Hg_*x*_Cd_1–*x*_Se NCs presented a technical challenge due to the susceptibility of the Hg-containing layer to interfacial alloying. Conventional shell growth techniques, typically involving Cd or Zn precursors in the form of metal oleates at temperatures exceeding 200 °C, led to a partial displacement of Hg from the Hg_*x*_Cd_1–*x*_Se layer. This resulted in widening of the QS band gap while obstructing the formation of a sufficiently thick shell necessary for an effective potential barrier at the surface. To address this issue, we employed a low-temperature approach using zinc diethyldithiocarbamate as a single-molecular precursor for ZnS shell deposition. This method, adapted from previous reports,^23^ allowed for a controlled shell growth at approximately 120 °C, leading to a substantial enhancement of the emission intensity.

TEM images of the CdS/Hg_*x*_Cd_1–*x*_Se core/shell and the fully shelled CdS/Hg_*x*_Cd_1–*x*_Se/ZnS QS structures are presented in Figure S1c,d, respectively. Comparison of the XRD patterns before and after Hg_*x*_Cd_1–*x*_Se shell growth in Figure 2a indicates that the Hg_*x*_Cd_1–*x*_Se shell semiconductor adopts a predominantly wurtzite structure, with Bragg reflections located between those of CdS and CdSe. Since bulk HgSe typically crystallizes in the zincblende phase, this intermediate peak position suggests a modest incorporation of Hg into CdSe, which does not alter the wurtzite crystallinity of the host. As seen in Figure S1d, the low-temperature ZnS shell deposition resulted in a somewhat strained shell structure, consistent with previous observations in ZnS-shelled nanoplatelets.^23^ Despite the strain-induced changes in the outer shell morphology, fabricated CdS/Hg_*x*_Cd_1–*x*_Se/ZnS QSs exhibited bright emission, with PL QY in the 30–60% range (see Figure 2b). In this study, we primarily targeted near-infrared spectral region, with PL wavelengths capped at approximately 1600 nm by keeping the shell thickness below ∼3 nm and using a Cd/Hg precursor ratio greater than 1. The corresponding evolution of the PL spectral position at various stages of CdS/Hg_*x*_Cd_1–*x*_Se/ZnS QSs synthesis in shown in Figures 1h and S1b. The observed spectral changes support the formation of the Hg-containing shell layer as well as the deposition of the ZnS shell.

We now turn to the dynamics of Auger recombination in QSs. Auger decay presents a significant challenge for lasing applications of zero-dimensional (0D) mercury chalcogenide QDs, as room-temperature biexciton lifetimes typically do not exceed 50 ps.^67^ For instance, Binks et al. reported biexciton lifetimes of approximately 49 ps for 3.5 nm HgTe QDs.^68^ Similarly, studies from Guyot-Sionnest, performed on larger HgTe QDs (5.4–7 nm in diameter), have placed biexciton lifetimes in the 40–50 ps range,^69^ while smaller HgTe QDs (nanoparticle diameter of 4–5 nm) exhibited biexciton lifetimes of 20–40 ps.^70^ Comparable trends have also been observed for HgSe QDs, where biexciton lifetimes fall within the 20–30 ps range.^71^

To estimate biexciton Auger lifetimes (τ_xx,Auger_) in CdS/Hg_*x*_Cd_1–*x*_Se/ZnS QSs, we first employed ensemble-averaged measurements using the variable-power PL intensity decay method.^42^ This approach, described in the SI Section 1, correlates the power-dependent PL intensity decay with the average number of absorbed photons per particle, < *N*_eh_>, assuming statistical scaling of multiexciton decay rates with < *N*_eh_>. Accordingly, τ_xx,Auger_ can be determined by fitting the measured PL decay traces at different excitation powers using numerical solutions of coupled rate equations (eq SE5). Here, the biexciton quantum yield (QY_*XX*_) is used as a universal fitting parameter across all decay traces. To this end, < *N*_eh_> was determined experimentally as < *N*_eh_> = *f*×σ, where *f* represents the laser pump fluence and σ is the absorption cross-section at the excitation wavelength. As shown in Figure 3a, the evolution of the PL intensity versus < *N*_eh_> = *f*×σ was fitted to the expected saturation curve, given by 1–e^–*f*σ^.^72^ Such a saturation of PL intensity at < *N*_eh_> > 1 occurs due to Auger recombination of multiexciton populations, making this fit most accurate when *QY*_*XX*_ ≪ 1. As an independent check of this analysis, we compared the extracted absorption cross-section at λ = 800 nm, σ = 4 × 10^–14^ cm^2^, with the approximate theoretical value obtained using Equation SE8, σ = 3.5 × 10^–14^ cm^2^. The observed discrepancy reflects the inherent uncertainty of this determination, arising from the approximate nature of the 1–e^–*f*σ^ fit and the assumptions underlying the theoretical model (see Supporting Information Section I). In addition to the excitation fluence, *f*, the model also incorporates the single-exciton lifetime as an input parameter (see eq SE4). Here τ_*x*_ = 105 ns was obtained from the low-power PL intensity decay measurement (Figure SF6b).

**Figure 3:**
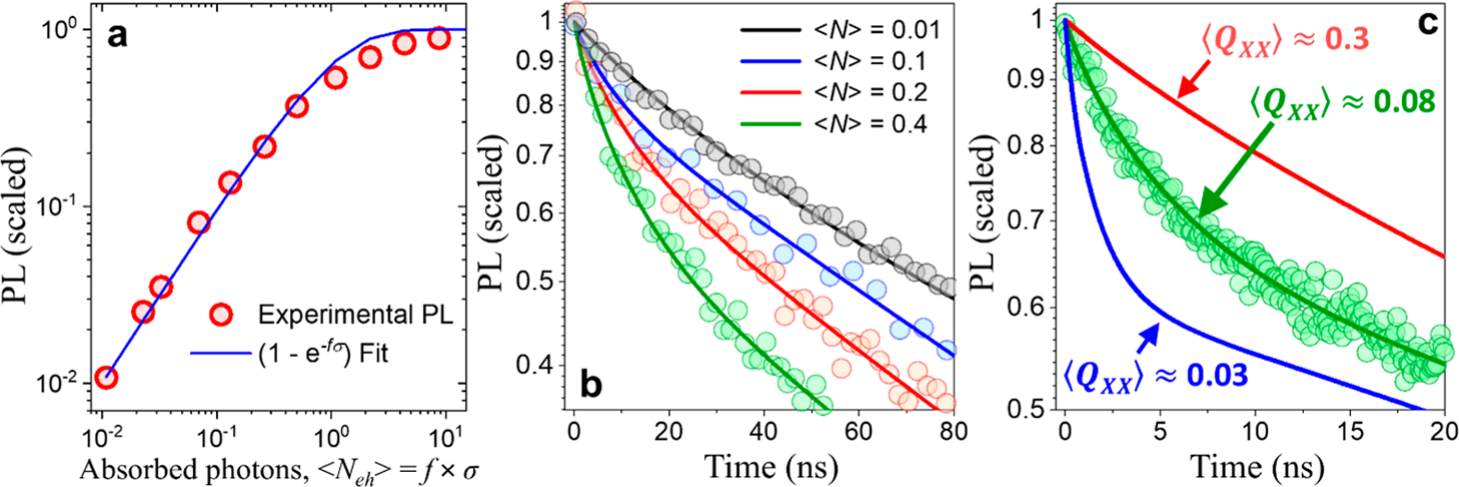
(a). The PL intensity as a function of excitation fluence is shown, with a theoretical fit applied using the model curve, 1–*e* ^–*f*σ^, to extract the absorption cross section, σ. (b). Time-resolved PL decay curves recorded for CdS/Hg_1–*x*_Cd_*x*_Se/ZnS QSs at several excitation fluences, corresponding to average exciton populations ⟨*N*⟩ ranging from 0.01 to 0.4. The decay profiles are modeled using a fitting parameter QY_*XX*_ = 0.08, capturing the dynamics of biexciton recombination and the suppression of nonradiative processes. (c). Comparison of experimental data at an excitation level of ⟨*N*⟩ = 0.4 with model fits using varying biexciton quantum yields. The three fits correspond to QY_*XX*_ = 0.03 (blue curve), QY_*XX*_ = 0.08 (red curve), and QY_*XX*_ = 0.3 (green curve), illustrating the sensitivity of the model to the suppression of nonradiative Auger processes.

Figure 3b illustrates the evolution of the PL intensity decay in CdS/Hg_1–*x*_Cd_*x*_Se/ZnS QSs as a function of increasing excitation power, corresponding to < *N*_eh_ > ranging from 0.01 to 0.4. All recorded PL­(t) curves were fitted using theoretical model predictions using eq SE5, with *QY*_*XX*_ as a single fitting parameter. The best fit was obtained for QY_*XX*_ = 0.08. For comparison, Figure 3c shows the PL intensity decay corresponding to QY_*XX*_ = 0.03 and 0.3, demonstrating the sensitivity of the model to variations in QY_*XX*_. Using the best fit to the experimental PL decay (QY_*XX*_ = 0.08), we then determined the biexciton Auger lifetime using

1 $$\tau_{2,Auger}=\frac{QYxx^{*}\tau_{X}}{\beta \left(1-QY_{xx}\right)QYx}$$

where τ_*x*_ is a single-exciton lifetime, and β represents the scaling of the biexciton radiative rate relative to that of single excitons. If all deexcitation paths involving the two electron–hole pairs are open, the statistical scaling of radiative rates with the number of electron–hole pairs, *m*, yields β = 4. Using eq 1, we estimated the biexciton lifetime in CdS/Hg_1–*x*_Cd_*x*_Se/ZnS QSs to be ∼8 ns, which is 160–180 times longer than in Hg_1–*x*_Cd_*x*_Se QDs with a similar emission range.^68,70^ Employing the same strategy for determining the biexciton lifetimes of CdS/HgS/CdS was not feasible due to the presence of the fast nonradiative component in the PL lifetime profile of these NCs, appearing even at low excitation powers (Figure 2c). Its interference with the fast decay of multiexciton populations made it difficult to discern the contributions of individual processes.

To gain deeper insight into higher-order exciton dynamics in QSs, we have carried out the femtosecond transient absorption (TA) spectroscopy measurements of both CdS/HgS/CdS and CdS/Hg_1–*x*_Cd_*x*_Se/ZnS samples. These tests were conducted across several independently synthesized batches to ensure reproducibility. For CdS/HgS/CdS QSs, the bleach recovery dynamics following excitation with an 800 nm pump pulse (pulse energy 9.3 μJ; (⟨*N*⟩ ≈ 19) are shown in Figure 4a. The bleach signal emerges within the instrument response time and decays on a subnanosecond time scale, with full recovery occurring in approximately 1000 ps. A relatively fast TA bleach recovery observed even at low excitation fluences (Figure 4c) agrees with the fast ∼2.0 ns decay component observed in PL lifetime measurements (Figure 2c). The initial excitation at 800 nm is localized in the HgS shell. These photoexcited carriers then partially delocalize into the surrounding CdS shell, effectively sampling a larger volume which reduces the effective confinement. This redistribution leads to a modest redshift of the bleach features over time, as seen in Figure 4a.

**Figure 4:**
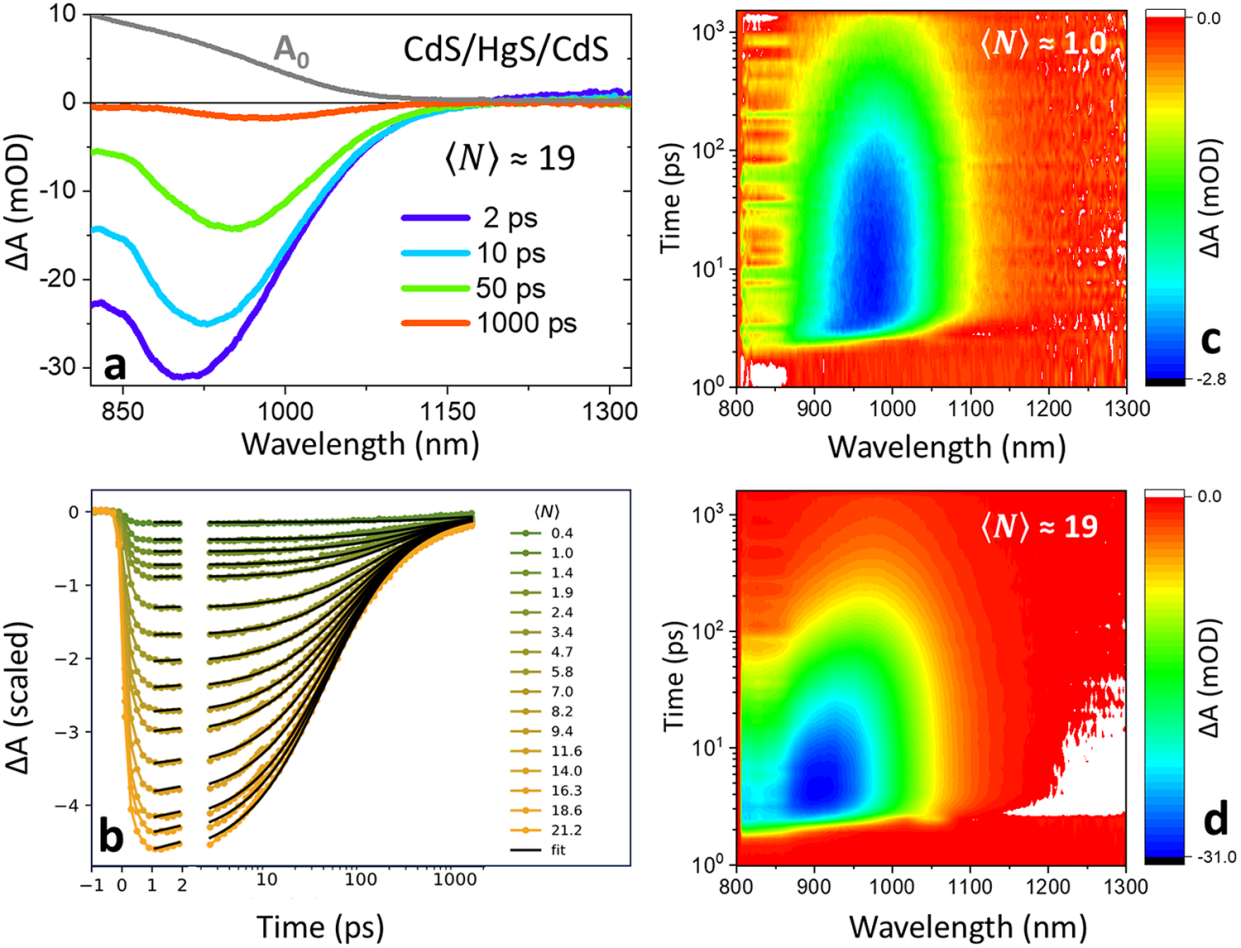
(a). Scaled linear absorption (*A*_0_) in arbitrary units and TA bleach recovery trace of HgS-based QSs (in units of mOD) following excitation with a pump pulse energy of 9.3 μJ, ⟨*N*⟩ = 19. (b). Spectrally integrated TA bleach recovery versus the excitation numbers, ⟨*N*⟩. (c) Two-dimensional Δ*A* map of HgS QSs recorded after excitation at 800 nm with a pump pulse energy of 0.5 μJ, corresponding to an average of ⟨*N*⟩ = 1.0 excitons per QS. (d) Δ*A* map of HgS QSs recorded under the same excitation wavelength with a higher pump pulse energy of 9.3 μJ, generating ⟨*N*⟩ ≈ 19 excitons per QS.

The spectrally integrated TA kinetics (Figure 4b) further demonstrate a continuous, fluence-dependent evolution of recombination dynamics, consistent with an increasing carrier density. This behavior is reminiscent of the CdSe based QS systems in the visible spectrum,^45^ where the decay was successfully modeled using a second-order recombination law

2 $$\frac{dn}{dt}=-k_{2}n^{2}$$

The model fit using the above eq 2 is represented in Figure 4b using black curves and describes biexciton recombination, where the decay rate increases with the square of the exciton population. In this regime, *k*_2_ plays the role of an effective two-particle recombination constant: larger *k*_2_ values correspond to more efficient exciton–exciton annihilation (e.g., via Auger processes), whereas smaller *k*_2_ indicate suppressed multicarrier decay. Solving eq 2 yields *n*(*t*) = *n*_0_/(1 + *k*_2_×*n*_0_×*t*), a characteristic nonexponential decay that describes the power-dependent PL dynamics. From a broader perspective, such kinetics imply that charge carriers (or excitons) recombine in a homogeneous energy landscape, where the recombination rate scales with both their interaction probability (*k*_2_) and their respective numbers (or better surface densities, *n* ^2^). Moreover, the absence of a first order (*k*_1_) term indicates that only a static offset is needed to capture lower order exciton state recombination. This observation is in-line with the multinanosecond decay of the XX states as asserted from the PL analysis above, i.e. *k*_1_ ≅ 0 for this analysis.

Figure 4c,d show the two-dimensional TA maps of CdS/HgS/CdS QSs (Δ*A* vs wavelength and delay time) for low (⟨*N*⟩ = 1.0; 0.5 μJ) and high (⟨*N*⟩ ≈ 19; 9.3 μJ) excitation pulse energies, respectively. In both cases, a strong ground-state bleach (950 nm), corresponding to band-edge excitons, appears following the photoexcitation. At higher excitation fluence (Figure 4d), the bleach amplitude increases and blue-shifts due to filling of higher energy states, while its recovery accelerates continuously with increasing *n*. The faster recovery is consistent with the presence of multiexciton states (high exciton density, *n*), which undergo more rapid decay due to a combination of enhanced multiexciton radiative recombination rates and accelerated nonradiative Auger processes at higher carrier densities. Overall, HgS-based QSs demonstrate a typical recovery of the excited state population, which mirrors the PL decay time scale at low density, with multiexciton contributions becoming increasingly significant under high-fluence excitation (⟨*N*⟩ ≫ 1).

Next, femtosecond TA spectroscopy was used to characterize the two representative CdS/Hg_1–*x*_Cd_*x*_Se/ZnS QS samples exhibiting different degrees of Cd/Hg intermixing within the quantum well layer. The corresponding two-dimensional TA maps are shown in Figure 5a–c. The first sample (HgCdSe_1550_) with the absorption edge at λ_gap_ = 1550 nm is characterized by a fully alloyed HgCdSe quantum well (Figure 5a,b), displays an excitonic bleach in the 1100–1550 nm region (also shown in Figure 5f). For a partly alloyed Sample HgCdSe_1350_ (λ_gap_ = 1350 nm) in Figure 5c, the excitonic bleach develops in the 600–1200 nm region (also shown in Figure 5e). The visible portion of the bleach is spectrally matched with the CdSe band edge absorption, suggesting a possible phase segregation within the HgCdSe shell. We speculate that the partial alloying has left the quantum shell separated into CdSe-rich and HgCdSe-rich domains, both contributing into the TA bleach.

**Figure 5:**
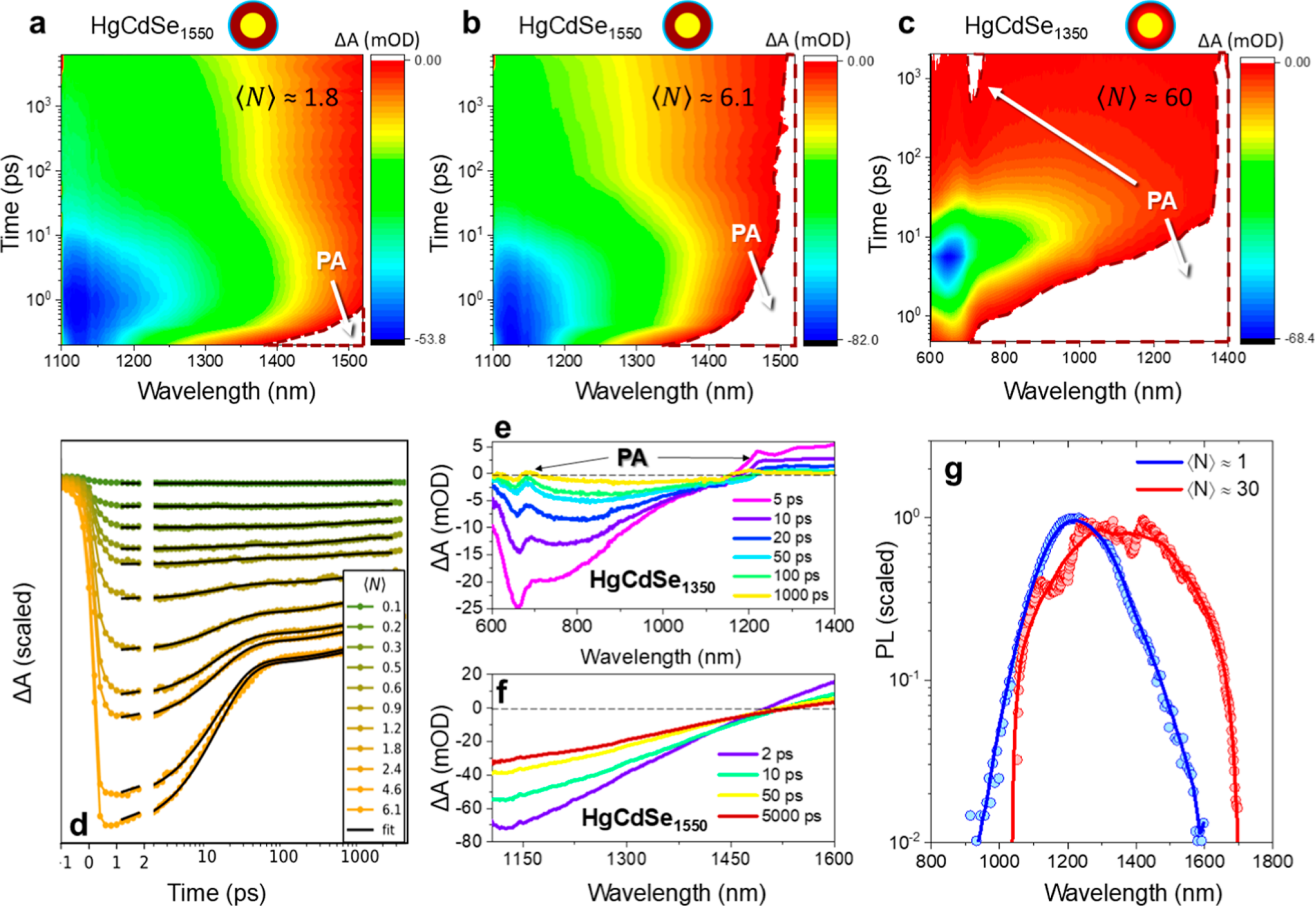
(a–c) Two-dimensional Δ*A* maps of HgCdSe-based QSs resulting from the 800 nm excitation. Corresponding linear absorptions are plotted in Figure S7. Panels (a,b) correspond to HgCdSe_1550_ sample (λ_gap_ = 1550 nm) excited using pump pulse energies of 17 μJ (⟨*N*⟩ = 1.8) and 40 μJ (⟨*N*⟩ = 6.1), respectively; panel (c) shows HgCdSe_1350_ sample (λ_gap_ = 1350 nm) under (⟨*N*⟩ = 60) excitation. Positive Δ*A* signals, corresponding to photoinduced absorption (PA), are highlighted in white. (d) Spectrally integrated TA bleach recovery versus the excitation numbers, ⟨*N*⟩. (e) TA bleach recovery trace for HgCdSe_1350_, showing two distinct PA features attributed to HgCdSe and CdSe band-edge states. (f) TA bleach recovery of HgCdSe_1550_, revealing a low-energy PA signal attributed to bound exciton states. (g) PL spectra of HgCdSe_1550_ under increasing excitation power, exhibiting redshift and spectral broadening.

Already at a first glance notable differences are observed between the HgS- and HgCdSe-based QS systems, both in temporal dynamics and the spectral content. Most notably, the HgCdSe-based systems show a much slower bleach recovery compared to HgS-based QSs, in agreement with the longer PL lifetimes observed in these materials (Figure 2d). A relatively slow recovery was attributed to the suppressed Auger recombination. This effect is clearly seen in the spectrally integrated TA recovery of a HgCdSe_1550_ sample, plotted on a linear scale (Figure 5d), where the estimated exciton occupancy ranges from ⟨*N*⟩ ≈ 0.1 at the lowest fluence to ⟨*N*⟩ ≈ 6.1 at the highest. For occupancies below ⟨*N*⟩ ≈ 1, the TA traces remain nearly flat, indicating the presence of only a small fraction of short-lived multiexciton states. Above this threshold, however, a pronounced fast decay component emerges, reflecting the acceleration of both radiative and nonradiative (Auger) recombination channels. The fast component appears as a discrete fixed recombination lifetime (or rate), different from the second order model observed for HgS QSs. Notably, the absence of this fast component at relatively low excitation fluences, ⟨*N*⟩ < 1, supports a nonlinear origin tied to high-order multiexciton dynamics, contrary to trapping of charges or excitons that manifest at low ⟨*N*⟩.

In case of HgCdSe-based QSs, the TA spectra of the two samples reveal an intriguing spectral feature: a long-lived photoinduced absorption (PA) band appearing on the red side of the band-edge bleach, well within the HgCdSe band gap. As shown in Figure 5a,b (highlighted in white), the PA signal grows with increasing pump pulse energy, which suggests a nonlinear intensity dependence. At low pulse energies (⟨*N*⟩ < 1), the PA signal vanishes entirely (Figure S8), confirming that its origin is related to multiexciton populations. This feature also displays unusual temporal characteristics. Unlike the fast-decaying PA commonly observed in colloidal QDs due to a transient Stark shift of energy levels,^73^ the PA feature in HgCdSe-based QSs persists on the nanosecond time scale. In the partly alloyed sample HgCdSe_1350_, the PA emerges not only in the near-infrared but also within the spectral region corresponding to the CdSe band gap (Figure 5c). It is possible that heat generated within these nanoparticles may dissipate into the surroundings on time scales exceeding 100 ps, potentially inducing transient phase changes, including bandgap narrowing.^74^ However, the higher-energy PA feature observed in HgCdSe_1350_ (λ = 700 nm) has a distinct onset delay of ∼300 ps following photoexcitation, suggesting a contribution from exciton or charge diffusion. Furthermore, according to TA spectra of HgCdSe_1550_ in Figure 5f, the low-energy PA persists for more than 5 ns, orders of magnitude longer than what has previously been reported for CdSe nanocrystals under comparable excitation densities.^74^

The observed low-energy PA feature is a clear signature of multiexciton states (⟨*N*⟩ > 1) with energies lower than that of an isolated exciton, which is indicative of exciton–exciton binding. Namely, the pump pulse causes two or more excitons to form a correlated complex whose total energy is then reduced due to binding. Such a negative exciton–exciton binding energy could mean the biexciton (or multiexciton) is a stabilized state, which absorbs at lower photon energies than the single-exciton transitions. Thus, once a single exciton is present in a particle, adding another exciton requires less energy, manifesting as a new PA peak red-shifted relative to the band-edge excitons, as illustrated in Figure 6a,b.

**Figure 6:**
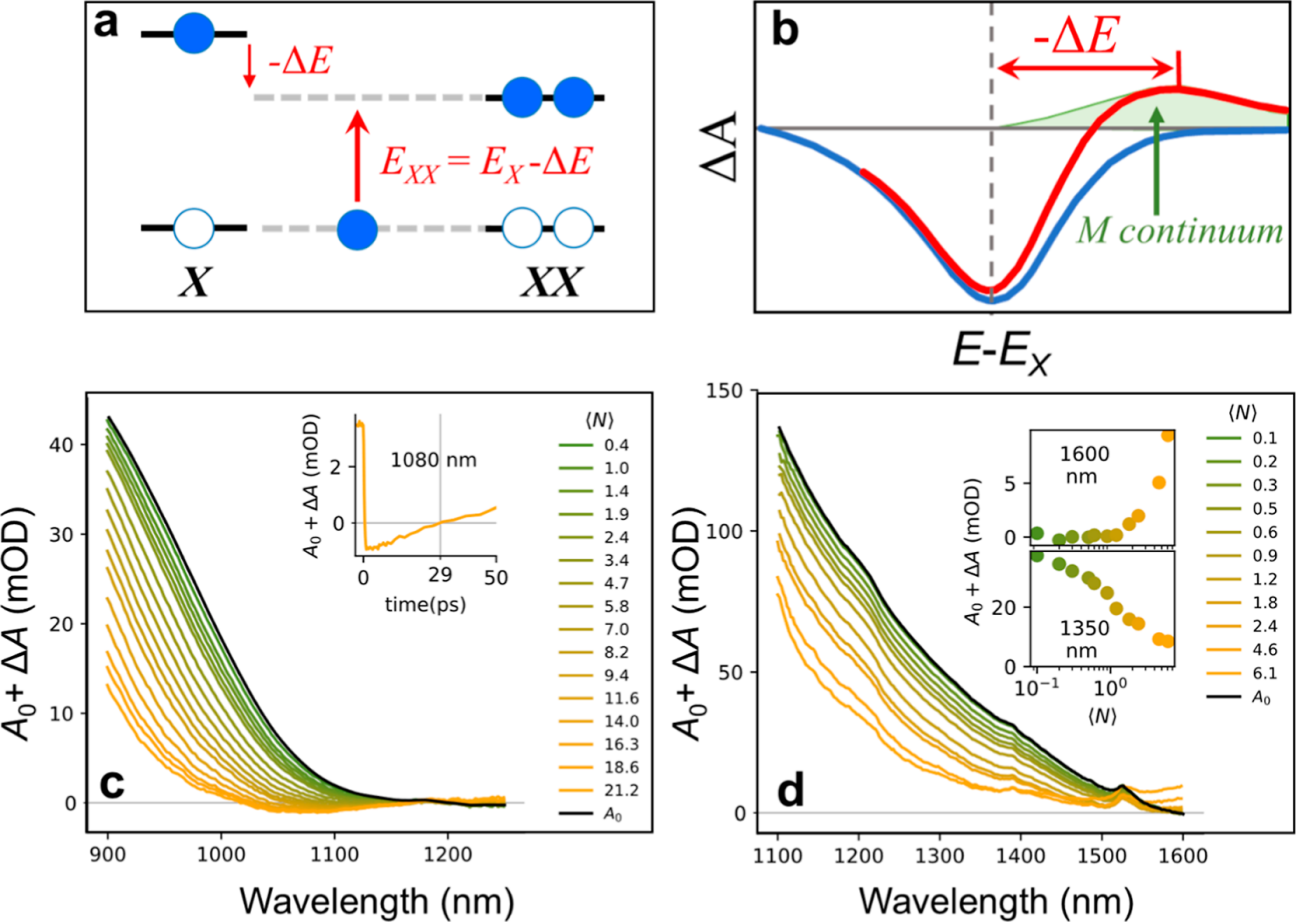
(a). Schematic energy diagram illustrating the ground-state exciton (X) and bound exciton complexes (XX, XXX, ...). Transitions from the ground state to the exciton (0 → X) and from the exciton to the exciton complex (-Δ*E*) are indicated. (b) Schematic illustration of the effect of bound exciton complexes on transient absorption spectra. The blue curve represents the scenario without excitonic binding, while the red curve shows the modified spectrum in the presence of excitonic complexes, highlighting the emergence of long-lived photoinduced absorption features (c). Optical gain spectrum of HgS-based QSs under femtosecond 800 nm excitation, exhibiting net stimulated emission in the 1025–1150 nm range. (d) Optical response of HgCdSe_1550_ QSs, showing no net optical gain, likely due to overlapping long-lived PA features.

The long-lived and spectrally distinct PA behavior in HgCdSe-based QSs is rarely seen in conventional colloidal NCs. Instead, it closely resembles signatures previously reported in epitaxial quantum wells at cryogenic temperatures,^49–51^ and in quasi-two-dimensional CdSe nanoplatelets at room temperature.^52,75^ In those systems, PA features have been attributed to the formation of exciton–exciton bound states, called excitonic molecules or quantum droplets.^75^ A similar trend is observed in HgCdSe-based QSs, where the delayed, long-lived PA signal suggests the presence of an exciton–exciton interaction channel that leads to the formation of bound exciton complexes. This interpretation is corroborated by the spectral redshift of the PA feature relative to the primary exciton bleach, most notably for the CdSe-associated PA band in HgCdSe_1350_ (Figure 5c and e), where the bleach–PA energy separation of ∼110 meV is comparable to the previously reported excitonic binding energy (Δ*M*) for CdSe nanoplatelets.^52^ Additional support for this assignment is provided by PL redshift observed at high excitation intensities (Figure 5h), which is consistent with exciton–exciton binding. Meanwhile, the concurrent broadening of the PL peak may reflect the coexistence of unbound excitons and bound exciton complexes. The formation of these exciton clusters implies strong attractive interactions between excitons in the HgCdSe-based QS architecture, sufficiently strong to support their coexistence with unbound excitons at room temperature, even at moderate carrier densities. To the best of our knowledge, this is the first experimental evidence of bound multiexciton states in near-IR 2D nanocrystalline systems.^76^

To evaluate the potential for optical gain in fabricated QSs, we examined the net absorption (*A* = Δ*A* + *A*_0_) under varying excitation fluences and at a delay of 5 ps after photoexcitation to allow for charge carrier cooling to complete. Optical gain is realized when the net absorption becomes negative (Δ*A* + *A*_0_ < 0), indicating that stimulated emission of the probe exceeds its absorption. Looking first at the HgS system (Figure 6c), we observe that the absorption is bleached away by state filling and no sizable spectral shifts are present. The absorption also stays zero at longer wavelengths indicating no photoinduced absorption takes place. At higher occupation (⟨*N*⟩ > 7), the total absorption becomes negative, indicative of net optical gain outcompeting absorption across the 1025–1150 nm spectral range. This gain is caused by a carrier density regime that is very different from the single or biexciton regime, in turn causing it to become substantially shorter lived. Nevertheless, the observed gain lifetime of 29 ps is comparable to the 20 ps gain observed in heavily doped PbS NCs (@ 1650 nm).^77^ Notably, achieving optical gain in colloidal infrared-emitting nanocrystals remains a significant challenge due to a rapid Auger recombination. Thus, the observation of net gain in the HgS-based QSs represents a promising step toward overcoming this limitation.

In contrast, CdS/Hg_*x*_Cd_1–*x*_Se/ZnS QSs did not produce a net optical gain under comparable excitation conditions, despite exhibiting a comparatively greater single and biexciton PL QYs. As shown in Figure 6d, the Δ*A* + *A*_0_ signal remained positive across the entire probe spectral range, indicating that photoinduced absorption dominates over stimulated emission in this system. The absence of the net gain can be attributed to the presence of long-lived competing PA feature, which grows in above ⟨*N*⟩ = 1 and ultimately suppresses stimulated emission. This PA signal extends to the edge of our detection window (1600 nm) and most likely extends much further. Subtracting the PA background plateau would result in net gain, indicating the system is capable of stimulated emission, but is counteracted by the PA.

The strong, fluence-dependent PA observed in the TA spectra of HgCdSe-based QSs can be explained by several mechanisms, one of which is the localization of charges or excitons. Notably, in this scenario, one would expect the localized potential wells to manifest already at the single-exciton level, producing a red-shifted or broadened PL band. However, the PL only red-shifts when ⟨*N*⟩ > 1 (see Figure 5g). Furthermore, the PA feature continues to grow as the occupancy increases beyond ⟨*N*⟩ ≈ 1 (Figure 6d), supporting the emergence of new transitions at progressively lower energies, which again is not consistent with multiexciton localization without bound complex formation.

Instead, the PA can be consistently explained by exciton–exciton binding into bound exciton states. In this framework, the addition of a second exciton to an already occupied nanostructure costs less energy than creating an isolated single exciton, leading to absorption at energies below the band edge. As exciton density increases, more biexciton and multiexciton complexes are formed, and the corresponding bound–state transitions produce the observed red-shifted PA. The persistence of absorption rather than gain (Figure 6d) reflects the relatively low amplitude of the negative signal (Δ*A*). Indeed, since PA amplitude exceeds linear *A*_0_ (Figure S7) in the 1500–1600 nm region, no net gain can be expected. Although the lack of optical gain appears unfavorable, the emergence of a strong PA component is noteworthy. This feature reflects the existence of low-energy excitonic states that may support nonlinear optical phenomena, such as exciton–biexciton cascade emission,^78^ single-exciton gain,^79^ or optical modulation^80^ in near-infrared colloidal nanocrystal systems.

Biexciton lifetime estimates resulting from variable-power PL intensity decay method above were corroborated by single-particle spectroscopy measurements. This approach allowed us to probe the dynamics of individual QSs with high temporal resolution, circumventing ensemble averaging effects that often obscure critical insights into biexciton behavior. By analyzing photon emission statistics and intensity fluctuations under controlled excitation conditions, we extracted characteristic signatures of single exciton and higher-order charged excitons. Figure 7a shows a characteristic PL time trajectory (blinking trace) of a CdS/Hg_1–*x*_Cd_*x*_Se/ZnS QS (HgCdSe_1350_, blue-side PL “tail” collected using Si photodiode with responsivity shown in Figure S9). Typically, blinking patterns show well-defined exciton and charged excitons (trions) intensity levels.

**Figure 7:**
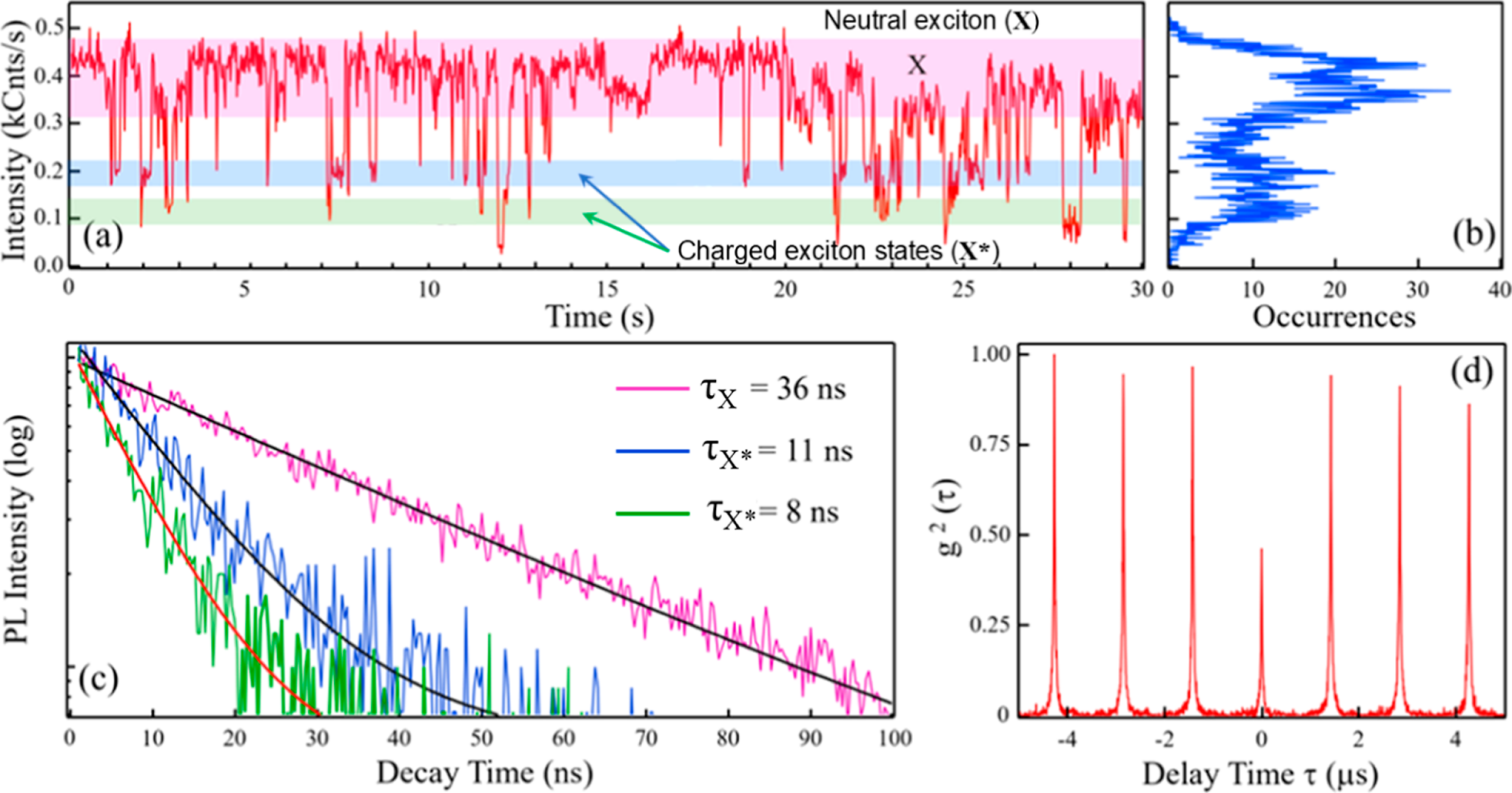
(a) A representative PL intensity time trace of CdS/Cd_*x*_Hg_1–*x*_Se/ZnS QSs, demonstrating characteristic blinking dynamics. The blinking trace shows discrete intensity levels corresponding to neutral excitons (X) and charged excitons (X*). The well-resolved intensity plateaus indicate the stability of these charge states and the minimal influence of Auger recombination, a hallmark of the quantum shell design. (b) The event occurrence histogram that shows distribution of intensity levels from panel (a). (c) Lifetimes associated with the X and X* intensity levels are extracted and presented as a color-coded plot consistent with the levels shown in panel (a). (d) Second-order correlation (g^2^(τ)) function for the single particle.

For particles in the bright state, nonradiative channels are strongly suppressed. So, during bright intervals the single-exciton QY is usually taken as ≈ 100%,^81–83^ and any reduction in intensity is attributed to additional nonradiative processes (charging, Auger, traps, etc.).^84^ In this framework, the highest PL intensity level is assigned to neutral single-exciton (X) emission, whereas lower-intensity levels (X*) are interpreted as being increasingly influenced by nonradiative relaxation channels and are often interpreted as charged exciton (trion-like) states, with possible contributions from higher-order charged multiexciton configurations.^85^ Using time-correlated single photon counting (TCSPC), we extracted emission lifetimes corresponding to different intensity levels observed in Figure 7a,b. For HgCdSe_1350_ sample, a representative single-particle trace reveals that the brightest emission state, attributed to the neutral exciton, follows a monoexponential decay with a lifetime of τ_X_ = 36 ns, while two dimmer levels, which we tentatively assign to negative and positive charged excitons, exhibit shorter lifetimes of τ^–^ = 11 ns and τ^+^ = 8 ns, respectively (Figure 7c). We then applied a previously established intensity–lifetime analysis,^86,87^ assuming these two charged states (labeled X*) correspond to X^–^ and X^+^ and using a simple superposition model for the biexciton Auger rate, $k_{A}^{XX}=2\left(k_{A}^{X^{-}}+k_{A}^{X^{+}}\right)$ ,^88^ to estimate biexciton radiative and Auger lifetimes and a corresponding biexciton PL QY of ∼39%. However, we emphasize that this biexciton QY estimate is not statistically robust, as it is derived from a single emitter and relies on speculative assignment of the dim states to specific trion configurations.

An independent measurement of QY_*XX*_ is possible via second-order correlation, the g^2^(τ) function. The ratio between the center peak (at delay time τ = 0) and the side peaks of the g^2^(τ) trace corresponds to the photon antibunching effect, indicating the probability of simultaneously detecting two photons within one excitation cycle. In case of a biexciton–exciton cascade, g^2^(0) = QY_*XX*_/QY_*X*_ in the limit of the weak pump (*N*≪1), as was first shown by Nair et al.^89^ Figure 7d presents g^2^(τ) function for the same dot that was used in the Auger lifetime analysis above. As seen, the value of the middle peak indicates QY_*xx*_ ∼45%, in agreement with the value computed using the trion lifetimes.

## Conclusions

In conclusion, this study establishes mercury chalcogenide quantum shells as a promising material class for near-infrared colloidal optoelectronics. By employing near-IR semiconductors with a two-dimensional quantum confinement geometry, we demonstrated that CdS/HgS/CdS QSs lead to the realization of optical gain and stimulated emission below 1150 nm. In parallel, the CdS/HgCdSe/ZnS QS system, despite exhibiting longer biexciton lifetimes and brighter emission compared to HgS-bases QSs, revealed an alternative ultrafast dynamic involving the formation of excitonic complexes. These bound excitons coexist with single excitons at room temperature introducing long-lived sub-bandgap absorption that competes with optical amplification. The presence of bound exciton complexes suggests that new physical regimes may be engineered. The ability to access and manipulate excitonic molecules at room temperature in near-IR offers a way toward novel photonic functionalities, such as biexciton–exciton cascade emission, optical modulation, or single-exciton gain.

## Methods

### Materials

The following chemicals were used as received without further purification or modification: anhydrous acetone (99%, Amresco), cadmium oxide (CdO, 99.95%, MilliporeSigma), Zinc acetate dihydrate­(98%, Acros Organics) anhydrous ethanol (99%, BeanTown Chemical), hexane (ACS grade, Thermo Scientific), 1-octadecene (ODE, technical grade, 90%, MiliporeSigma), Octane (98%, MiliporeSigma), 1-octanethiol (97%, Alfa Aesar), oleic acid (OA, technical grade, 90%, MiliporeSigma), oleylamine (OLAM, technical grade, 70%, MiliporeSigma), Dioctylamine­(DOA, 97%, MiliporeSigma), rhodamine 101 inner salt (R101, 94%, pure, Thermo Scientific), selenium powder (Se, 99.5%, 200 mesh, Thermo Scientific), sulfur powder (S, 99.999%, Thermo Scientific), toluene (99.8%, MiliporeSigma), and tir-*n*-octylphosphine (TOP, 97%, Strem Chemical).

### Synthesis of CdS “Core” Nanocrystals

CdS NCs (“bulk” size) were synthesized according to a previously published coalescence-based growth procedure.^61^ In brief, 8 mL OLAM and 42 mg CdCl_2_ were loaded into a 25 mL flask and put under an argon atmosphere (maintained using a Schlenk line) and heated to 290 °C. Once the temperature was stabilized, 540 nmols of small CdS seed NCs (2–4 nm diameter) were swiftly injected into the flask and left to coalesce for 60 min. The flask was then removed from the heating mantle and quenched in a water bath after it cools below 220 °C (this avoids cracking the glass with the heat shock). The NCs were cleaned via precipitation with toluene/ethanol (∼1:2) mixture. The gray precipitate was removed by redissolving the pellet in toluene and precipitating it out with a centrifuge, leaving a transparent yellow solution that is cleaned with another toluene/ethanol mixture. The NCs were then dissolved in a mixture of 5 mL of ODE and 8 mL of OA and loaded into a 25 mL flask. The flask was again put under the argon atmosphere and heated to 150 °C for 60 min and then cooled to room temperature and precipitated as before. The final NCs were suspended in hexane and stored under ambient conditions. This produces NCs with a (large) diameter of 12–16 nm. NCs with a (giant) 16–20 nm diameter were synthesized with an identical procedure except the temperature was raised to 320 °C and 1080 nmols of small CdS seeds were injected.

### Synthesis of CdS/HgS/CdS Quantum Shells

In a 25 mL three-neck flask under inert atmosphere, HgCl_2_ (80 mg), oleylamine (OLAM, 5 mL), and 1-octadecene (ODE, 1 mL) were heated to 110 °C with stirring until HgCl_2_ dissolved; large CdS nanocrystals (150 nmol in 1 mL ODE) were then swiftly injected, the temperature was set to 95 °C, and the reaction was held until the desired band-edge emission wavelength was reached. Octanethiol (OT, 0.2 mL) was added and the mixture reacted for 2 min before cooling to <40 °C and isolating the product by EtOH/toluene centrifugation, yielding CdS/HgS (∼1 ML thickness). The solids were redispersed in OLAM (1 mL) and ODE (1 mL), heated to 50 °C, and 0.1 M S-ODE was injected dropwise until the band-edge emission disappeared, corresponding to ∼1/2 ML S^2–^ surface coverage. The temperature was raised to 120 °C and 0.1 M Hg­(OAc)_2_-OLAM was injected for the same amount of time; subsequent growth of the HgS shell was achieved by simultaneous coinjection of S-ODE and Hg­(OAc)_2_-OLAM at 120 °C until the desired emission wavelength was obtained. After cooling to room temperature and purification with EtOH/toluene, the nanocrystals were redispersed in 5 mL of 0.1 M Cd­(OA)_2_ and flash-heated to 320 °C, followed by a cleaning step and redispersion in OLAM (1 mL) and ODE (1 mL); the dispersion was reheated to 260 °C for growth of the final CdS protective shell by dropwise coinjection of 0.1 M Cd­(OA)_2_ and S-TOP at 2.5 mL/h, after which the mixture was cooled and purified.

### Synthesis of CdS_bulk_-CdHgSe-ZnS Quantum Shells

CdSe shell growth on CdS core nanocrystals was carried out by syringe-pump coinjection of two separate precursors −0.1 M Cd-oleate and 0.1 M TOP–Se (not premixed). The Cd-oleate precursor was prepared by charging a 50 mL flask with CdO (412 mg), oleic acid (OA, 8 mL), and 1-octadecene (ODE, 5 mL), heating to 260 °C under argon until a clear, nearly colorless solution formed, then injecting an additional 19 mL ODE. The Se precursor was prepared by combining selenium powder (141 mg) with trioctylphosphine (TOP, 3 mL) in a 25 mL flask and heating to 140 °C under argon until all selenium reacted, followed by dilution with 14 mL ODE. For shelling, 540 or 1080 nmol of CdS NCs were loaded into a 100 mL flask with oleylamine (OLAM, 3 mL) and ODE (3 mL), heated to 100 °C, and degassed until bubbling ceased; the flask was then placed under argon (Schlenk) and the temperature set to 315 °C. At 270 °C, the Cd and Se precursors were introduced at 3 mL h^–1^ and continued until the emission reached the desired wavelength (typically 630–680 nm), with total injection times of ∼120 min for giant NCs and ∼90 min for large NCs. The reaction was removed from the heating mantle, cooled to room temperature, cleaned by precipitation with a toluene/ethanol mixture, and the final CdS/CdSe NCs suspended in hexane. Mercury was then incorporated into the CdSe layer by cation exchange: HgCl_2_ (25 mg) and OLAM (4 mL) were heated to 80 °C until dissolved, ODE (1 mL) was added, and CdS/CdSe particles (dispersed in 2 mL ODE) were injected at 70 °C; the mixture was raised to 80 °C and held 5–7 min, precipitated with ethanol and a little acetone, and redispersed in hexane. For subsequent shelling, the CdS/CdHgSe particles (in hexane), ODE (1 mL), and OLAM (3 mL) were combined in a round-bottom flask, held under vacuum for 5–10 min at 70 °C, and zinc diethyldithiocarbamate (0.1 M in TOP) was introduced via syringe pump at 80 °C; the temperature was then raised to 120 °C during injection and the reaction maintained at 120 °C for 2 h.

### Characterization

Absorbance spectra were acquired using a Cary 60 scan spectrophotometer. Photoluminescence (PL) spectra were measured by excitation with a pulsed 405 nm laser diode (PDL 800-D, Picoquant) and emitted photons were collected by a fiber optic cable connected to an Andor Shamrock 303i spectrograph and measured using an Andor Newton 970 EMCCD. PL decay dynamics were acquired via 405 nm pulsed excitation (Picoquant) and photons were detected with an ID100–50 single photon detector (ID Quantique) and processed by a SPC-130 TCSPC module (Becker & Hickl). Transmission electron microscope (TEM) images were acquired at 200 kV on carbon films coated with a copper grid (300 mesh, obtained from Electron Microscope Science) using a Thermo Fisher Talos F200X G2 S/TEM. Relative quantum yield (QY) measurements were acquired with a 532 nm CW DPSS laser (MeshTel) and R101 dye was used as a reference (99% QY in ethanol). Absolute photoluminescence quantum yields were measured using Quantaurus-QY Absolute PL quantum yield spectrometer C11347 (Hamamatsu, Inc., Japan)

### Transient Absorption Measurements

The Ghent University transient absorption measurements where performed on a home-build setup based on a Yb/YAG laser (Light Conversion, Pharos). The 170 fs pulses at 1030 nm where split into a pump and probe path. The pump is converted to 800 nm via an optical parametric amplifier (Light Conversion, Orpheus) and excites the sample at a 0.5 kHz repetition rate. The probe is generated by focusing the laser pulse into a 10 mm YAG crystal to generate a supercontinuum. For the measurements on HgS QS, we used the 800 nm light to generate a probe between 800 and 1300 nm. The measurements on HgCdSe QS use a probe between 1150 and 1500 nm generated with the 1030 nm seed. The samples were dispersed in an infrared transparent solvent (TCE) and continuously stirred during the measurement. The pump spot size is measured with a beam profiler (Thorlabs, BC106N–UV).

The BGSU femtosecond transient absorption spectrometer used in these experiments is based on a regeneratively amplified Ti/sapphire laser system (Solstice Ace, Spectra-Physics: 800 nm, 95 fs, 4.8 mJ pulse^–1^, and 1 kHz). The output was split 25:75. The 25% portion was used in these experiments, which was further split 50:50. The 0.41 mJ pulse^–1^ portion was taken from the first half and routed to pump an optical parametric amplifier (TOPAS-C, Light-Conversion) to generate 370 nm excitation light pulses (forth-harmonic-signal). The 370 nm output was chopped at 500 Hz using a mechanical chopper, and sent through a Berek compensator to set the polarization plane to the magic angle (54.7°) with respect to that of the probe light to eliminate signals from rotational dynamics of the solute, and then focused onto the sample. The excitation energy at the sample position was attenuated using variable neutral density filters. The typical excitation energy used was ∼1 mJ pulse^–1^. Either a broadband white-light continuum (wlc) in the 600–1140 nm range or a single-wavelength TOPAS-C output tunable from 930 to 1400 nm were used for probing. For the wlc probing, the remaining half of the 25% portion was routed into another TOPAS-C tuned to produce the signal output at 1200 nm. This output was sent through a variable time-delay stage, attenuated, and then focused onto a 4 mm thick CaF_2_ window to generate the wlc beam. The latter was split into a probe and a reference beam. The reference beam, which is used as a reference for the correction of shot-to-shot intensity fluctuations of the wlc pulses, bypassed the sample. The probe beam was focused onto the sample using the fully reflective optics and overlapped with the excitation beam at a 6° angle at the sample position, with the excitation and probe beam diameters being 120 and 75 μm (full width at half-maximum, fwhm), respectively. Afterward, the probe and reference beams were dispersed by a spectrograph (Acton SP 2358), and the corresponding light was recorded in a 274 nm window using two 512-pixel diode arrays (Hamamatsu S8380–512Q). Cut-off filters were placed in front of the spectrograph entrance slit to eliminate second-order diffraction of the white-light continuum. For the single-wavelength probing, the same TOPAS-C was tuned to produce the variable output from 930 to 1400 nm in 20 nm increment steps (signal, ≥1100 nm; second-harmonic-idler, <1100 nm). This output was attenuated, sent through the same delay stage, and then steered into the same optical path toward the sample as that of wlc. For the TOPAS-C probe, the probe and reference beams were detected by a pair of matching Si or Ge photodiodes. For both wlc and TOPAS-C probing, the detectors were synchronized to the 1 kHz repetition rate of the amplified laser system and read out at 1 kHz for derivation of transient absorption (Δ*A*) signals. Specifically, the difference between the decadic logarithms of a probe-to-reference intensity ratio at a certain probe wavelength and a certain delay stage position acquired for two consecutive laser shots, where for one the excitation is chopped (off) and for another the excitation excites the sample (on) is the transient absorption signal at the given delay time and the probe wavelength: $\Delta A=-\log_{10}{\left(\frac{I_{\mathrm{pr}}}{I_{ref}}\right)}_{on}-\left(-\log_{10}{\left(\frac{I_{\mathrm{pr}}}{I_{ref}}\right)}_{off}\right)$ . This signal was averaged using 300 pairs of consecutive laser shots before moving to the next delay time and is further averaged over 5 to 15 delay line scans to yield the reported Δ*A* data point.

The sample solutions were kept in a 2 mm path length spinning cell made of CaF_2_. All measurements were done using fresh solutions at 21 °C. All Δ*A* data were corrected for either group-velocity dispersion in the wlc probe light or different arrival times of the TOPAS-C single-wavelength components with a 30 fs accuracy using Pascher Instruments AB software (Lund, Sweden). The cross-phase modulation signals due to electronic instantaneous response of the solvent (shaped as two negative troughs flanking a positive crest) centered at a time zero between excitation and probe pulses^90,91^ were exploited for the correction. Using these signals, the cross-correlation function (CCF) between the excitation and probe pulses is evaluated to be Gaussian shaped with the width (fwhm) of 120 fs. The Δ*A* signals from neat solvent (*n*-hexane) were measured immediately after transient absorption experiments on the sample. These solvent Δ*A* signals persisted for the initial 300 fs and were negligibly small compared to the solute Δ*A* signals, and therefore, were not further considered in the analysis of the solute dynamics.

### Single-Dot Spectroscopy

A concentrated solution of g-QSs in hexane was diluted and deposited onto a glass substrate with an approximate surface density of less than 0.01 per μm^2^. The sample was mounted on a translation stage of an optical microscope and excited with 405 nm, 50 ps pulses through a 100×, 1.2 NA oil-immersion objective that is also used to collect PL. The laser repetition rate is set between 1 and 2 MHz, ensuring that the time between pulses is significantly longer than the PL decay times, allowing complete relaxation of excitons between successive excitations. The emitted PL signal is directed to a pair of PerkinElmer avalanche photodiodes (SPCM AQR-13) arranged in a standard Hanbury–Brown–Twiss configuration with a 50/50 beam splitter. Time-tagged, time-correlated single-photon counting (TCSPC) is carried out using PicoQuant MultiHarp 150 electronics, which simultaneously record photon arrival times relative to both the start of the measurement and each laser excitation pulse. This enables the construction of PL decay profiles for selected time intervals within the PL intensity trace or specific windows within the intensity distribution. Custom software developed in IGOR is used to extract photon streams from each detector channel and compute gated g^2^(τ) functions at any TG value for biexciton quantum yield determination.

### IR Emission and PL Intensity Decay

IR-range steady-state and time-resolved PL measurements were conducted on a home-built spectroscopy platform. For steady-state PL, samples were excited with a 445 nm continuous-wave laser; the emission was dispersed by a monochromator and detected with a low-noise photoreceiver, with a long-pass filter inserted to suppress residual excitation light. Time-resolved PL was acquired using a time-correlated single-photon counting (TCSPC) system in which the emission was spectrally resolved by a monochromator and detected with photomultiplier tubes spanning the visible (160–900 nm) and near-infrared (950–1700 nm) ranges. Excitation for TCSPC was provided by a picosecond fiber laser, yielding an overall instrument response of ∼200 ps.

## Supplementary Material



## Notes

Spectroscopy characterization of QSs, additional TEM images, rate equations and simulations (PDF)
