# Supplementary material for: Bound Exciton Complexes in Near-Infrared Emitting Quantum Shells
Source: ACS Nano. 2026 Jan 15;20(4):3776–90. doi: 10.1021/acsnano.5c18640 (PMC12874638; doi:10.1021/acsnano.5c18640)
Supplement: Supplementary file 1 [file nn5c18640_si_001.pdf]

Supporting Information for:

Bound Exciton Complexes in Near-Infrared Emitting Quantum Shells

Dulanjan Harankahage[1,2] Divesh Nazar,[1,2] Korneel Molkens,[5,6,8] Mykhailo V. Bondarchuk,[1,3] Christopher M. Hicks,[1,3] Andrew A. Marder,[4] Michael Montemurri,[2] Adam Roach,[1,2] Ivo Tanghe,[5,6] Liangfeng Sun,[1,2] Richard D. Schaller,[7,9] Benjamin T. Diroll,[7] Anton V. Malko,[4] Alexander N. Tarnovsky,[1,3] Dries van Thourhout,[8] Zeger Hens, [5,6] Pieter Geiregat,[5,6] Mikhail Zamkov[1,2*]*

The Center for Photochemical Sciences,[1] Department of Physics,[2] and Department of Chemistry,[3] Bowling Green State University, Bowling Green, Ohio 43403, USA

Department of Physics, The University of Texas at Dallas, Richardson, TX, 75080, USA[4]

Physics and Chemistry of Nanostructures, Ghent University, 9000 Gent, Belgium[5]

NOLIMITS Center for Non-Linear Microscopy and Spectroscopy, Ghent University, 9000 Gent, Belgium[6]

Center for Nanomaterials, Argonne National Laboratory, Lemont, IL, 60439, USA[7]

Photonics Research Group, Department of Information Technology, Ghent University, 9000 Gent, Belgium[8]

Department of Chemistry, Northwestern University, Evanston, IL 60208, USA.[9]

Corresponding author: Pieter.Geiregat@UGent.be; Tel: +32 9 264 48 64

Corresponding author: zamkovm@bgsu.edu; Tel: 419-372-0264; Fax: 419-372-9938

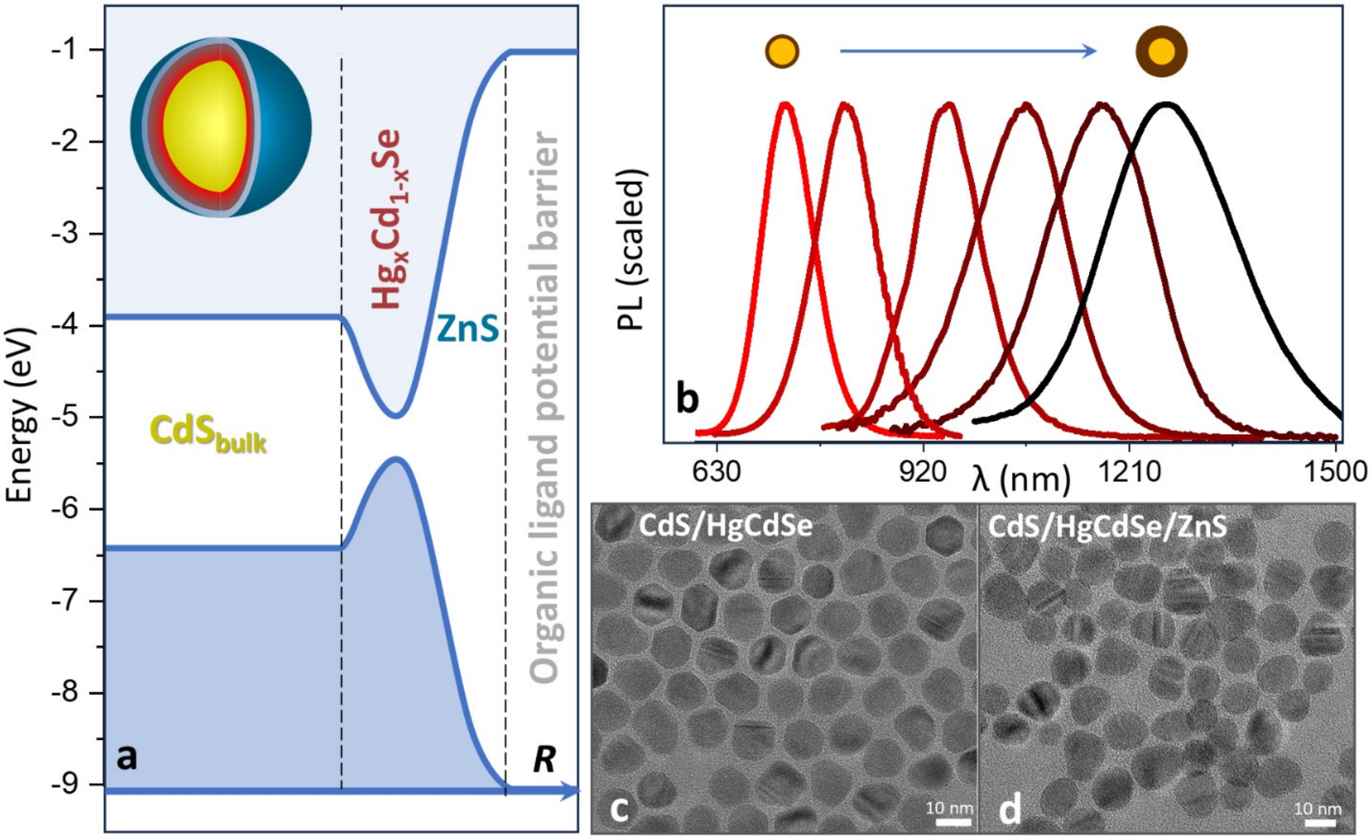

Figure S1. (a). Schematic illustration of the conduction (light blue) and valence (dark blue) bands of CdS/$Hg_xCd_{1-x}Se$/ZnS QSs showing the formation of a $Hg_{1-x}Cd_xSe$ quantum well confined between wide-bandgap CdS and ZnS layers. The outer material depicts the large effective barrier associated with the organic ligand shell, which is grown over a thin, 1-2 monolayer ZnS shell. (b). Photoluminescence peak position as a function of the $Hg_xCd_{1-x}Se$ shell thickness, demonstrating tunable emission wavelengths across the near-infrared spectral range. (c,d) Transmission electron microscopy (TEM) images showing the effect of shelling on nanocrystal morphology: (d) intermediate CdS/$Hg_{1-x}Cd_xSe$ core/shell structures, grown over CdS core particles (shown in Fig.2b of the main text) and (e) finished CdS/$Hg_xCd_{1-x}Se$/ZnS QSs.

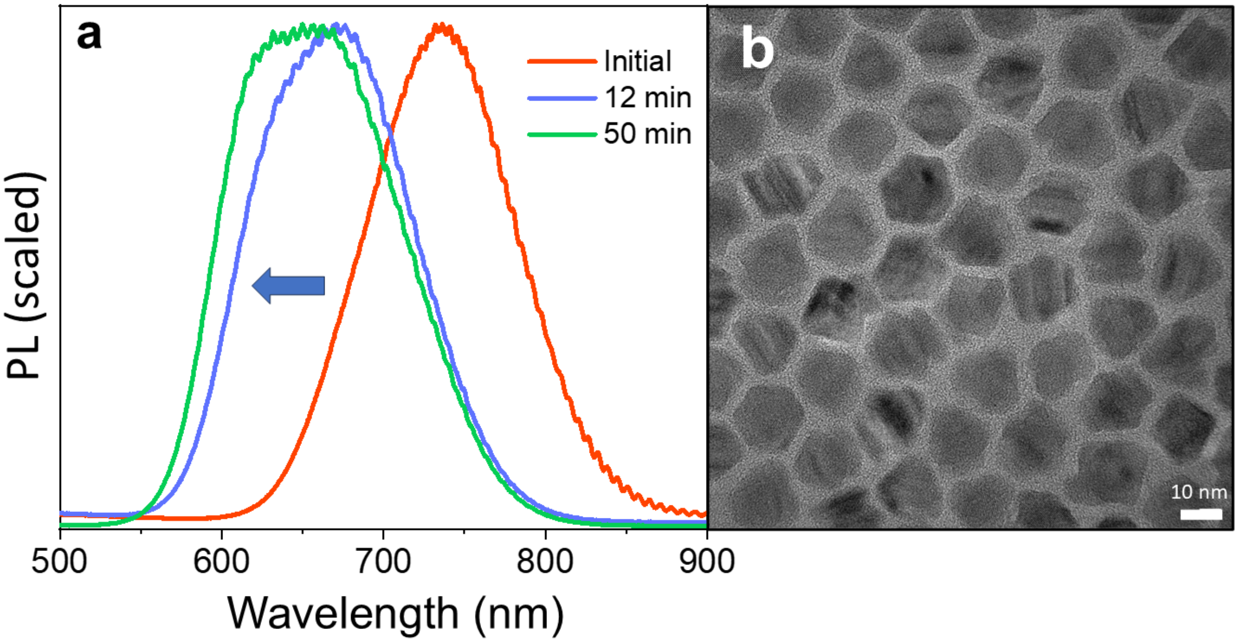

Figure S2. Phase transition from β-HgS to α-HgS shell layers upon heating to 290°C in ODE. (a) The emission of CdS/HgS/CdS undergoes a blue shift to 630 nm, corresponding to the bandgap of α-HgS. (b) TEM image of α-HgS, highlighting the significant lattice strain at the HgS/CdS interface.

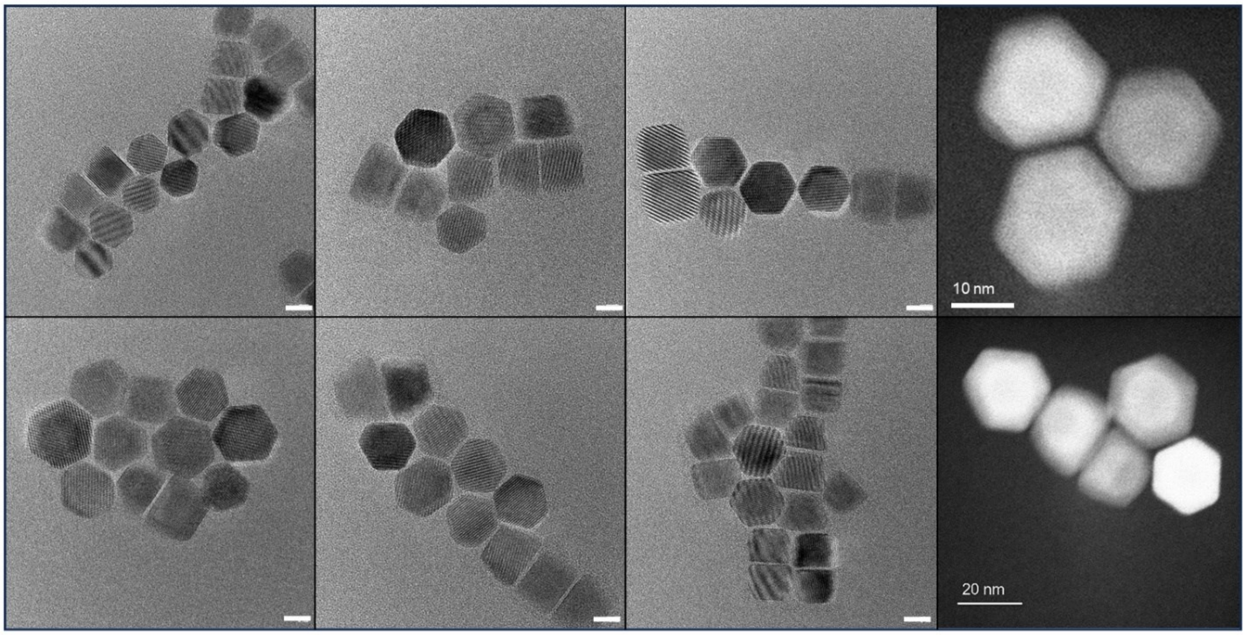

Figure S3. TEM and HAADF-STEM images of CdS/HgS/CdS quantum shells, illustrating the impact of annealing on transforming the nanoparticle shape into a hexagonal morphology.

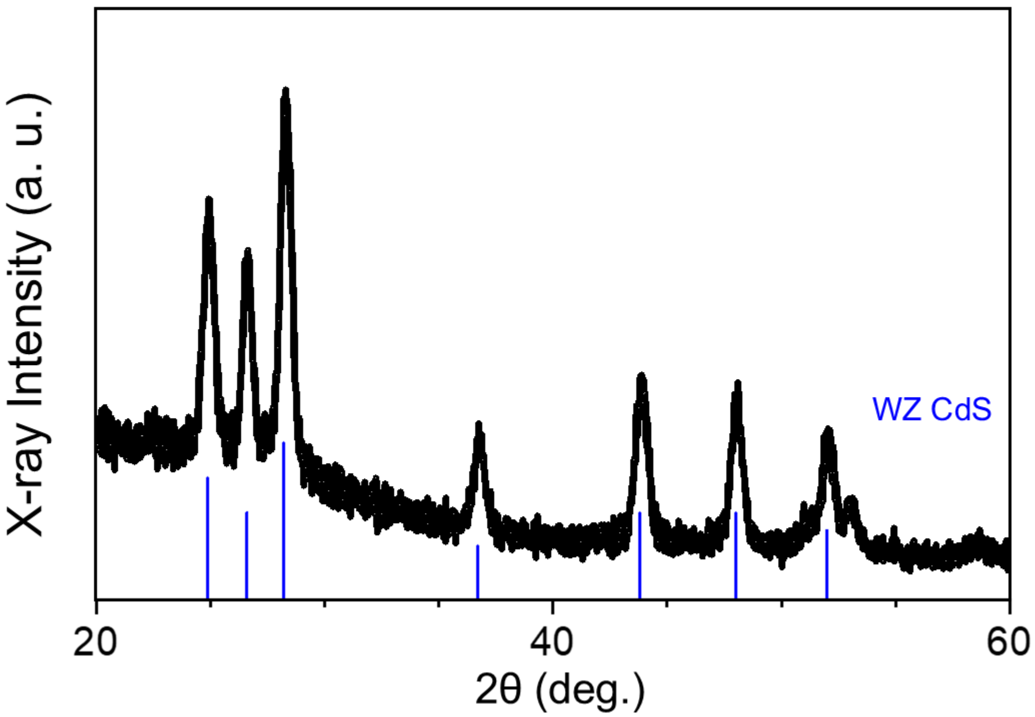

Figure S4. XRD pattern of CdS/HgS/CdS QSs showcasing distinct diffraction peaks corresponding to WZ CdS crystal structure.

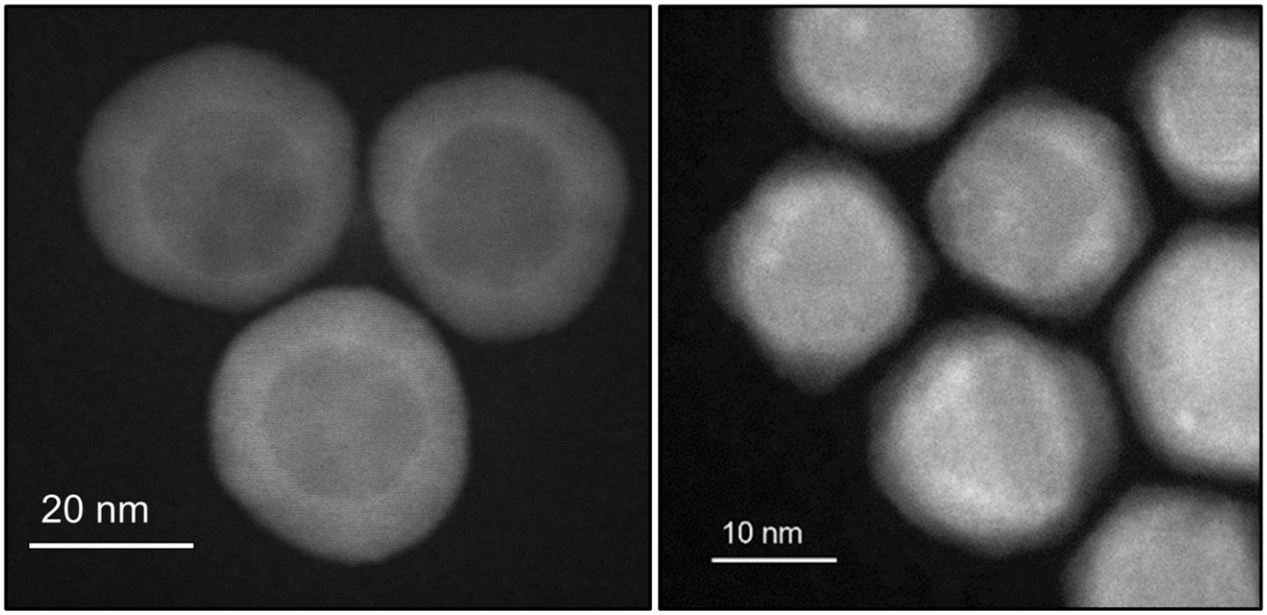

Figure S5. STEM images of CdS/HgS/CdS quantum shells, highlighting the effects of excessive CdS shell growth, which leads to unintended alloying at the HgS/CdS interfaces.

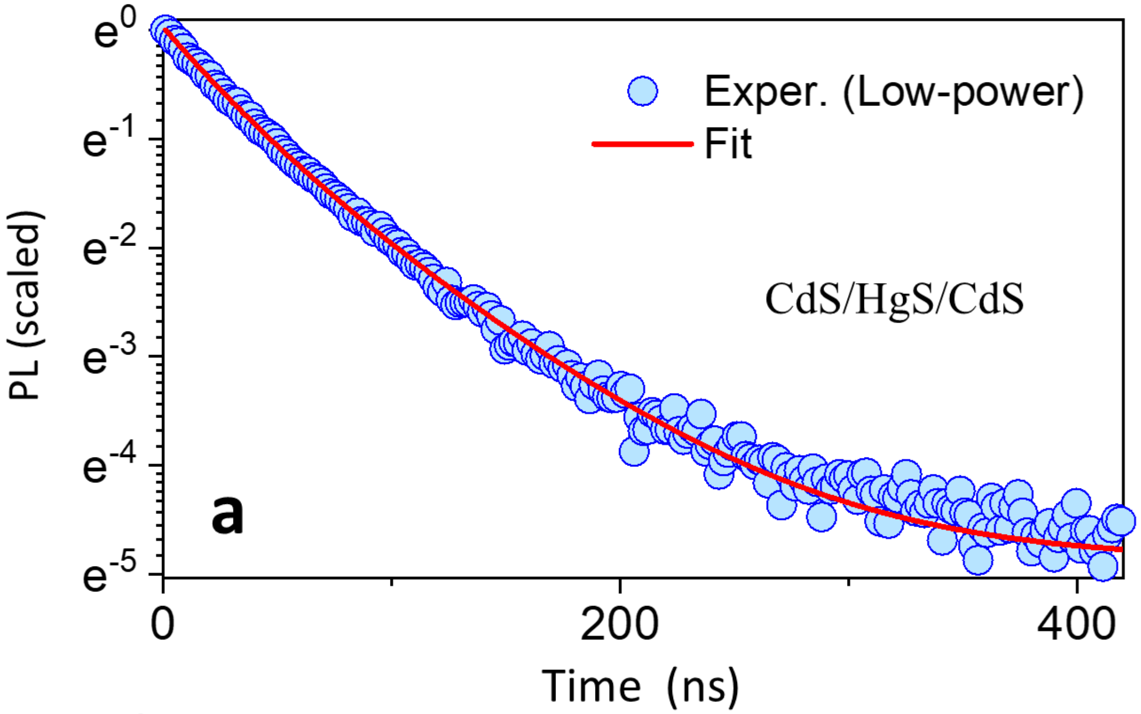

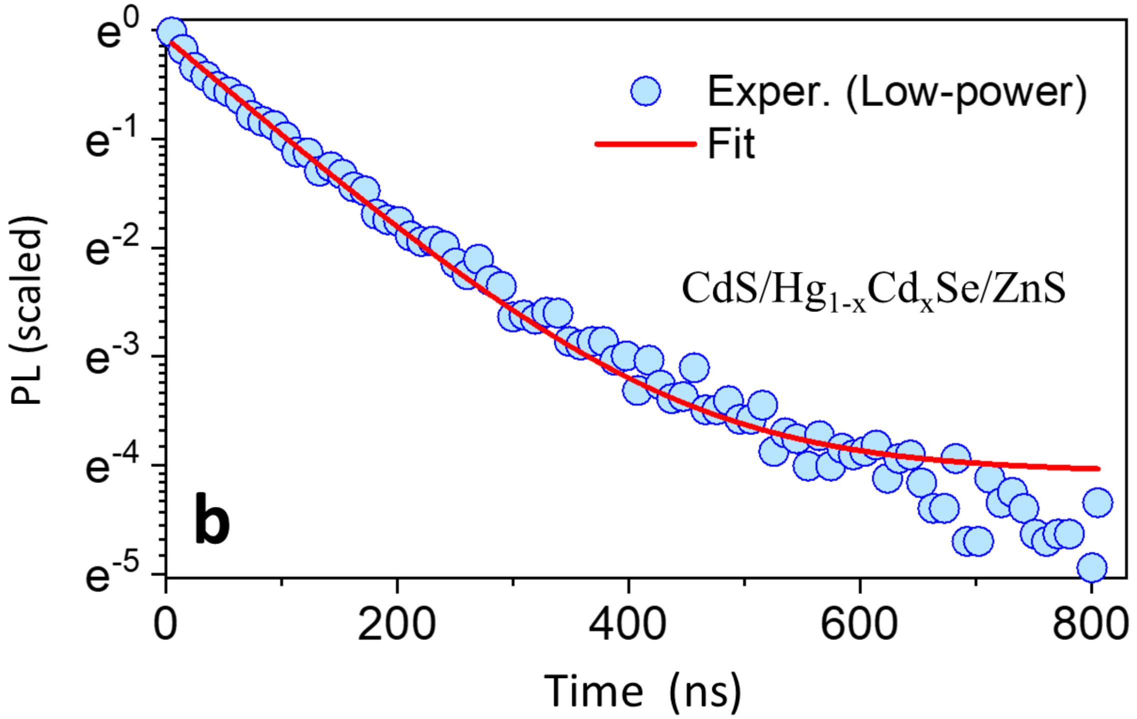

Figure S6. (a) PL intensity decay of CdS/HgS/CdS QSs under low-power excitation, corresponding to a thin-shell HgS sample (PL peak at λ = 820 nm). Experimental data is fitted using a double exponential decay. (b) PL intensity decay of $CdS/Hg_{1-x}Cd_xSe_{1550}/ZnS$ QSs under low-power excitation. Experimental data is fitted with Aexp(-τ/105 ns) + B.

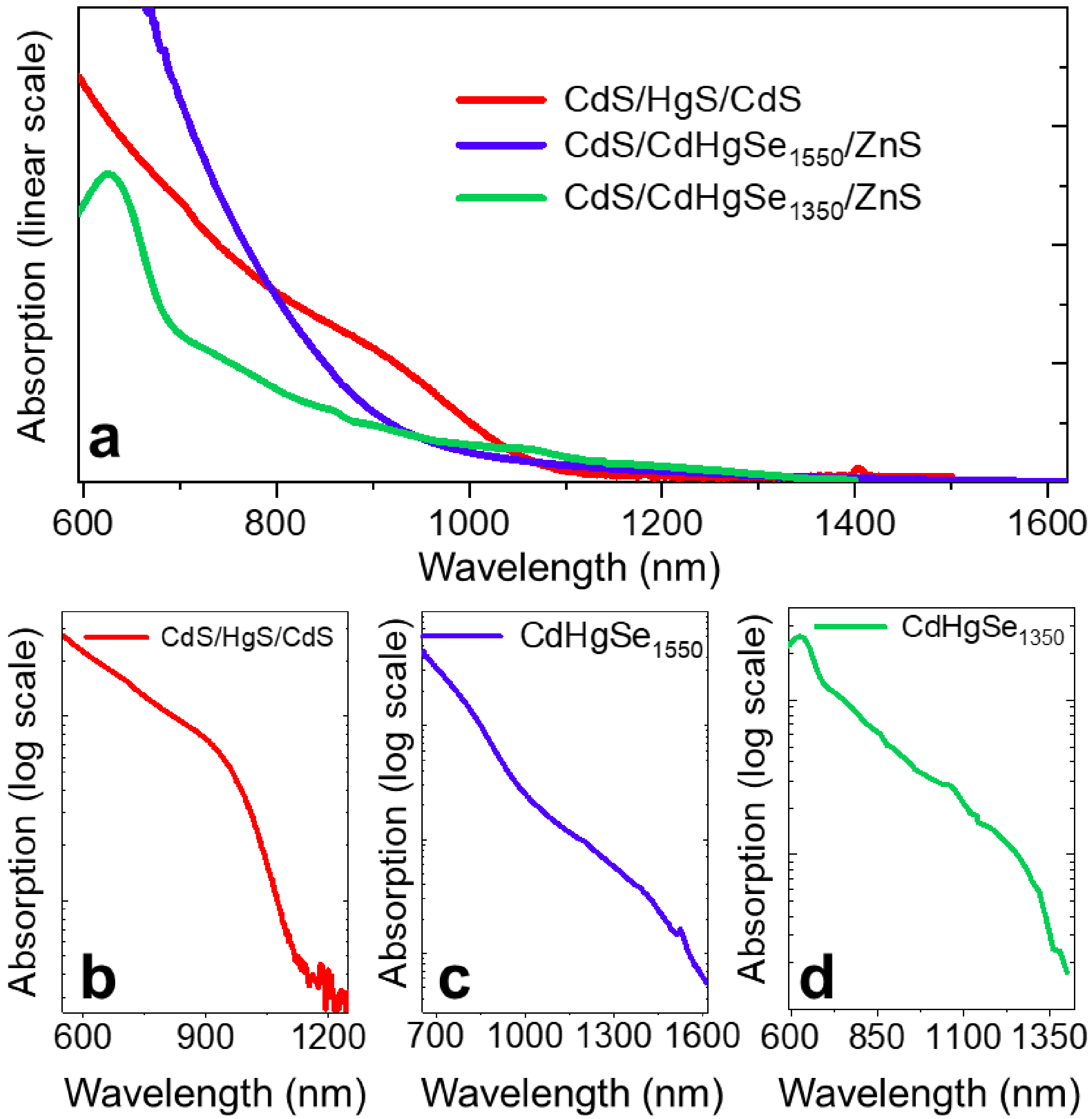

Figure S7. (a) Linear absorption of CdS/HgS/CdS (red), CdS/$CdHgSe_{1550}$/ZnS (blue), and CdS/$CdHgSe_{1350}$/ZnS (green), plotted on a linear scale. Log10 scale absorption of CdS/HgS/CdS in (c), CdS/$CdHgSe_{1550}$/ZnS (d), and CdS/$CdHgSe_{1350}$/ZnS in (d).

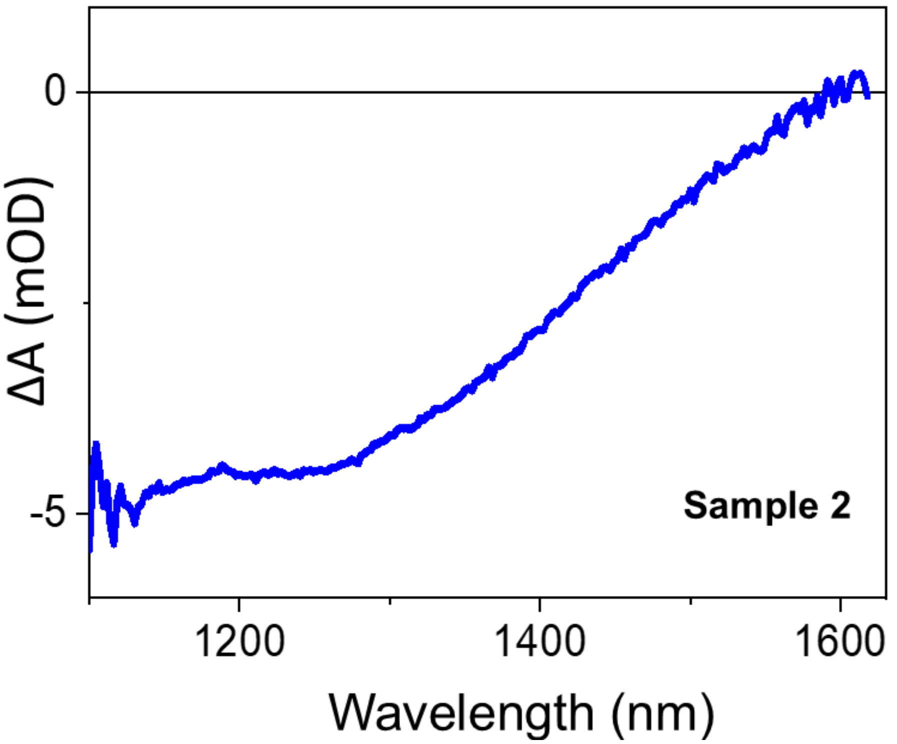

Figure S8. (a). TA bleach recovery trace of $HgCdSe_{1350}$-based QSs following excitation with a low pump pulse energy of 0.6 μJ. No photoinduced absorption is observed. Pump-probe delay = 5 ps.

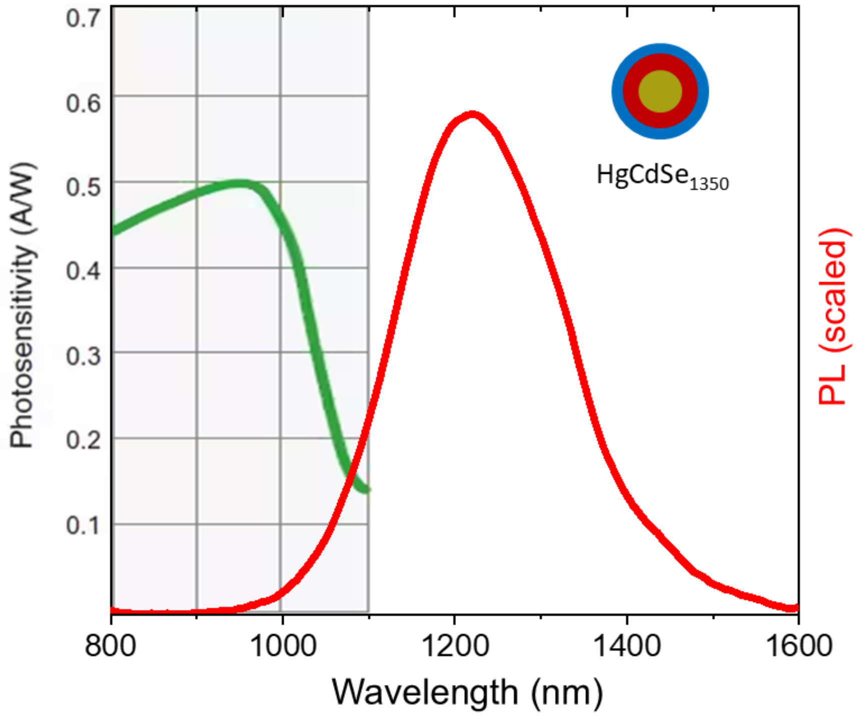

Figure S9. Comparison of the silicon photodiode responsivity with the emission spectrum of the $HgCdSe_{1335}$ sample indicating an overlap in the 950 – 1150 nm region.

Section I. PL intensity decay fitting procedure:

The experimental PL intensity decay in Figures 4b and 4c of the main text was compared to the model calculations of PL emission from a multi-exciton state. The model estimated the time evolution of PL for a given average number of excitation photons per particle, $<N_{eh}>$, with biexciton emission quantum yield, QY_{XX}, being the only fitting parameter. The value of $<N_{eh}>$ was first used to determine the initial multiexciton population distribution using the Poisson formula:

$$f(m) = \langle N_{eh} \rangle^{m} \times e^{-m} / m! \tag{SE1}$$

where m is the number of excitons per particle.

For each excitonic state m, we can then determine the radiative ($k_{m,r}$) and nonradiative ($k_{m,nr}$) decay rates using statistical scaling of exciton decay rate by Klimov et al., [1]:

$$k_{m,r} = \mathrm{m}^2 k_{r,2}/4, \qquad k_{m,nr} = \mathrm{m}^2(\mathrm{m}-1)\, k_{nr,2}/4 \tag{SE2}$$

where $k_{r,2}$ and $k_{nr,2}$ are the corresponding radiative and nonradiative rates for m = 2 populations (biexcitons). In this work, these rates were determined using a fitting parameter, QY_{xx}, and known values of a single-exciton total decay constant, $\tau_{tot,1}$, and the single-exciton state PL QY, QY_X:

$$k_{2,r} = 2^2\, \mathrm{k}_{r,1} = 4 \times \mathrm{QY_x}/\tau_{tot,1} \tag{SE3}$$

$$k_{2,nr} = 4 \times \frac{1-QY_{xx}}{QY_{xx}} \times k_{1,r} \;=\; 4 \times \frac{1-QY_{xx}}{QY_{xx}} \times \frac{QY_x}{\tau_{tot,1}} \tag{SE4}$$

Time-dependent population dynamics were then computed using a finite-difference time-step approach, accounting for radiative decay, nonradiative decay, and cascading transitions between adjacent exciton states. Considering that Auger decay of a m-exciton state results in a state with (m - 1) excitons, the temporal evolution of the m-exciton population in a QS, $P(m, t)$, is then determined by solving coupled rate equations:

$$\frac{dP(m,t)}{dt} \;=\; k_{m+1}P(m+1,\, t) - k_m P(m,\, t) \tag{SE5}$$

where, $k_m = k_{m,r} + k_{m,nr}$, represents the total decay rate of an m-exciton state.

The time-dependent PL emitted by a particle ensemble with an average number of excitons of $<N_{eh}>$ is thus given by:

$$PL_{total}(N\_eh, QY\_XX) = \sum_{m=1}^{Nmax=60} m \times P(m,t) \tag{SE6}$$

The total PL emitted by a particle ensemble with an average number of excitons of <N_{eh}> is thus given by:

$$PL_{total} = \int_0^{\infty} \sum_{m=1}^{Nmax=60} m \times P(m,t)\ dt \qquad \text{(SE7)}$$

Section II. Determination of absorption cross-section from the TEM images for QSs:

The absorption cross-section, σ of g-QSs was determined using the formula:

$$\sigma_{\omega} = \frac{4\pi\alpha\omega}{n_{medium}} |f(\omega)|^2 R^3$$

where:

- σ_{ω} = absorption cross section at angular frequency ω
- α = fine structure constant $(\frac{e^2}{\hbar c} \approx \frac{1}{137})$
- ω = angular frequency of the incident light (in rad/s)
- $f(\omega)$ = oscillator strength of the electronic transition at frequency ω
- n_{medium} = refractive index of the surrounding medium
- R = radius of the nanocrystal

This expression can be simplified as:

$$\sigma_{\omega} = \xi(\omega) R^3$$

where $\xi(\omega)$ is a material and transition-specific constant that incorporates the optical matrix elements and environmental parameters. For instance, in the case of 12-nm CdS/CdHgSe/ZnS QSs featuring 8.5-nm CdS core, the cross section was calculated as:[2]

$$\sigma_{1.5eV} = 1.62 \times 10^{-16} \times R^3(nm)[cm^2] \qquad \text{(SE8)}$$

References.

[1] Klimov, V. I.; McGuire, J. A.; Schaller, R. D.; Rupasov, V. I. Scaling of Multiexciton Lifetimes in Semiconductor Nanocrystals. *Phys. Rev. B* **2008**, *77* (19), 195324.

[2] Guzelturk, B.; Diroll, B. T.; Cassidy, J. P.; Harankahage, D.; Hua, M.; Lin, X.-M.; Iyer, V.; Schaller, R.D.; Lawrie, B.; Zamkov, M. Bright, Fast, and Durable Scintillation from Colloidal Quantum Shells. *Nature Comm.* **2024**, *15*, 4274.
